# Analysis of proteasome stress in different cell types defines a conserved transcriptional response signature and its modulation with aging

**DOI:** 10.1093/g3journal/jkag245

**Published:** 2026-09-16

**Authors:** Mamta Rai, Yong-Dong Wang, Anna Stephan, Anjana Nityanandam, Fabio Demontis

**Affiliations:** Department of Developmental Neurobiology, St. Jude Children's Research Hospital, Memphis, TN 38105, United States; Department of Cell and Molecular Biology, St. Jude Children's Research Hospital, Memphis, TN 38105, United States; Department of Developmental Neurobiology, St. Jude Children's Research Hospital, Memphis, TN 38105, United States; Department of Developmental Neurobiology, St. Jude Children's Research Hospital, Memphis, TN 38105, United States; Department of Developmental Neurobiology, St. Jude Children's Research Hospital, Memphis, TN 38105, United States

**Keywords:** proteasome, stress response, proteostasis, aging, stress signature, organoids

## Abstract

The proteasome is essential for proteostasis. Transcriptional induction of proteasomal components occurs when the proteasome is inhibited, but an overview of the transcriptional responses caused by proteasome perturbation is missing. Here, we profiled transcriptional changes caused by chemical and genetic proteasome inhibition and defined time–dose responses in cells and organoids. Induction of proteasome components varied by cell type and inhibition mode, whereas other responses were consistent, including upregulation of chaperones and secreted factors, and repression of cell cycle regulators. A proteasome stress response signature was defined based on the genes consistently modulated across systems, and applying this signature to aging datasets revealed activation of this stress response in some tissues, including skeletal muscle. Moreover, secreted factors within the signature showed similar age-related changes in human plasma, suggesting systemic activation of this stress response with aging. Together, these findings define a transcriptional signature for monitoring proteasome stress during aging and age-related diseases.

## Introduction

The proteasome is a fundamental system for cell homeostasis (Bedford et al. 2011; Schmidt and Finley 2014; Collins and Goldberg 2017). In concert with the ubiquitination cascade, the proteasome ensures regular protein turnover and the degradation of misfolded proteins that accumulate in response to damage and stress (Bedford et al. 2011; Schmidt and Finley 2014; Collins and Goldberg 2017). While proteasomes are located in the cytoplasm and nucleus, they also mediate the degradation of proteins extracted from other organelles, such as endoplasmic reticulum proteins, exported to the cytoplasm via the Endoplasmic-Reticulum-Associated Degradation (ERAD) pathway (van den Boom and Meyer 2018), and from multimolecular assemblies, such as the sarcomere (Piccirillo and Goldberg 2012).

The activity of the ubiquitin-proteasome system is influenced by many intracellular and environmental factors (Bedford et al. 2011; Schmidt and Finley 2014; Collins and Goldberg 2017); therefore, stress responses have evolved to cope with the perturbation of different components of this cascade (Rai et al. 2025b). Ubiquitin depletion triggers a ubiquitin stress response characterized by the transcriptional upregulation of ubiquitin (Finley et al. 1987) and changes in the proteasome composition and deubiquitinase activity that increase ubiquitin salvage and restore the free ubiquitin pool (Hanna et al. 2007; Kimura et al. 2009). Perturbation of the early steps of the ubiquitination cascade (knockdown of the main E1 ubiquitin-activating enzyme UBA1 or concomitant knockdown of multiple E2 ubiquitin-conjugating enzymes) leads to a different adaptation, which preserves peroxisomal protein import (a process which relies on the cyclic ubiquitination of the cargo receptor Pex5) via the decreased degradation (and hence upregulation) of peroxisomal proteins of the import docking complex (Hunt et al. 2023).

Previous studies in yeast and other models demonstrated that direct inhibition of the proteasome elicits a backup response known as the proteasome stress response (Bush et al. 1997; Lee and Goldberg 1998). While earlier studies highlighted the induction of chaperones in response to proteasome inhibition (Bush et al. 1997; Lee and Goldberg 1998), subsequent studies indicated that a key element of the proteasome stress response is the increased expression of proteasome components (Xie and Varshavsky 2001; Lundgren et al. 2005; Steffen et al. 2010; Radhakrishnan et al. 2014; Sha and Goldberg 2014; Koizumi et al. 2016; Lehrbach and Ruvkun 2016; Lehrbach et al. 2019; Dirac-Svejstrup et al. 2020), which can compensate for the decline in proteasome function by providing the basis for the assembly of new proteasomes (Lee et al. 2004; Hanssum et al. 2014). In addition, alternative proteolytic systems are also activated, ie autophagy (Kageyama et al. 2014; Sha et al. 2018; Tsakiri et al. 2019) and proteases (Glas et al. 1998; Geier et al. 1999; Wang et al. 2000; Lipinszki et al. 2013; Rai et al. 2022; Jiao et al. 2023).

Physiologically, proteasome stress may occur in conditions of proteostatic challenges, whereby the proteasome degradatory capacity is overwhelmed by the accumulation of misfolded polyubiquitinated proteins (Dantuma and Lindsten 2010) or by conditions (such as exercise) in which muscle contraction mechanically damages contractile proteins (Close et al. 2005; Morton et al. 2009; Arndt et al. 2010; Ogborn et al. 2014). Several pathological conditions are also known to affect proteasome function (Bedford et al. 2011; Schmidt and Finley 2014; Collins and Goldberg 2017; Chocron et al. 2022) and hence may trigger a proteasome stress response. For example, pathogenic aggregation-prone proteins such as mutant tau can clog the proteasome and impair its function (Keck et al. 2003; Tseng et al. 2008). Moreover, proteasome inhibitors such bortezomib are used in the clinic to treat multiple myeloma and other types of cancer (Kisselev and Goldberg 2001; Goldberg and Rock 2002; Obeng et al. 2006; Goldberg 2012; Kamens et al. 2023), and the induction of the proteasome stress response may constitute an important component of these treatments.

Despite the notion that the proteasome stress response increases the expression of proteasome components (Rai et al. 2025b), relatively few studies have provided a genome-wide analysis of the transcriptional changes induced by proteasome inhibition. Therefore, it remains unknown whether other gene categories are key and widespread outputs resulting from perturbation of the proteasome across diverse cell systems.

Recent studies in vivo suggest that the proteasome stress response may have pervasive effects on local and systemic proteostasis not limited to the expression of proteasome components but also resulting in the compensatory upregulation of proteases, chaperones, and secreted factors that regulate proteostasis (Rai et al. 2021a; Rai and Demontis 2022; Rai et al. 2025a). Despite these recent studies, a thorough characterization of the proteasome stress response is missing.

Previous studies for other stress responses defined transcriptional signatures via the global analysis of stress-induced changes across multiple cell types (Murray et al. 2004; Han et al. 2008; Kelley et al. 2025; Wang et al. 2025). For example, a transcriptional signature of cell senescence was established by interrogating the responses induced by exposing different cell types to triggers of cell senescence (Casella et al. 2019; Saul et al. 2022). While these studies have greatly advanced the analysis of cell senescence and in particular the interrogation of whether cell senescence occurs in different physiological and pathological contexts (Casella et al. 2019; Saul et al. 2022), a similar signature is missing for the proteasome stress response, thus limiting the understanding of its occurrence in normal and disease contexts. To address this gap in knowledge, here, we have examined proteasome stress in human HEK293T cells, mouse C2C12 myotubes and myoblasts, and human brain organoids by perturbing proteasome function with the chemical inhibitor MG132 and/or via the siRNA-mediated knockdown of components of the catalytic core of the proteasome.

We find that modulation of proteasome gene expression generally increases upon proteasome inhibition but varies depending on the specific proteasome component and cell system considered. Notably, proteasome stress leads to upregulation of chaperones and secreted factors, and downregulation of cell cycle regulators. Moreover, from these analyses with diverse tools and in distinct cell types, we identify a transcriptional signature of proteasome stress and utilize this gene set to define the occurrence of the proteasome stress response during aging across mouse tissues and organs and systemically in humans. We propose that these new resource datasets may help foster studies on the proteasome stress response and monitor its occurrence in diverse contexts.

## Methods

### HEK293T cell culture and transfection

Human HEK293T cells (ATCC #CRL-3216; authenticated and verified to be mycoplasma-free) were maintained at 37 °C with 5% CO_2_ in high-glucose Dulbecco′s Modified Eagle Medium (DMEM) containing Glutamax (Gibco #10566016), supplemented with 10% fetal bovine serum (FBS; Gibco #10437-028) and penicillin–streptomycin (Gibco #15140122). Cells were seeded into 12-well plates at ∼50,000 cells per mL. Transfection was performed once cultures reached ∼80% confluence (typically the following day) using protocols similar to those previously used (Hunt et al. 2023, 2025b).

For gene silencing, HEK293T cultures were treated with a SMARTpool mix of 4 siRNAs targeting the same gene (Dharmacon), ie either PSMA3 siRNAs (#L-011758-00-0050), PSMA4 siRNAs (#L-017211-00-0050), PSMB2 siRNAs (#L-010461-00-0050), PSMB5 siRNAs (#L-004522-00-0050), or non-targeting (NT) control siRNAs (#D-001810-10-50). In each well, 50 pmol siRNA per µL Lipofectamine was mixed with 50 µL OptiMEM and combined with 1 mL of culture medium. For transfection with DNA plasmids (CMV > EGFP and CMV > Fluc^DM^-EGFP, Addgene #90172; cytomegalovirus promoter (CMV); enhanced green fluorescent protein (EGFP), 1 µg of DNA was combined with 2 µL of Lipofectamine, which was then added to 50 µL of OptiMEM and then to 1 mL of culture (for each well of a 12-well plate). The cells were analyzed 3 d after transfection.

### C2C12 myoblast culture and siRNA transfection

C2C12 mouse myoblasts (ATCC #CRL-1772; verified to be mycoplasma-free) were grown at 37 °C with 5% CO_2_ in high-glucose DMEM with Glutamax (Gibco #10566016), supplemented with 10% FBS (Gibco #10437-028) and 1% penicillin–streptomycin (Gibco #15140122) (Hunt et al. 2015; Hunt et al. 2019a; Graca et al. 2022; Stephan et al. 2022). Myoblasts were then transfected with siRNAs (either gene-specific or NT controls) using Lipofectamine 2000 (Invitrogen #11668027) at a ratio of 2 µL Lipofectamine to 50 pmol siRNA in OptiMEM (Gibco #31985062). The ON-TARGETplus siRNAs (Dharmacon) were the following: either NT control siRNAs (#D-001810-50), Psmb2 siRNAs (#L-062847-01-0050), or Psmb5 siRNAs (#L-043960-01-0050). The cells were analyzed 3 d after transfection. For the analyses of myotubes, C2C12 myoblasts were differentiated with 2% horse serum-containing media (Gibco, #26050070), as previously done (Hunt et al. 2015; Hunt et al. 2019a; Graca et al. 2022; Stephan et al. 2022).

### Generation of human brain organoids

Human brain (cortical) organoids were generated from H9/WA09 cells (WiCell; NIH approval NIHhESC-10-0062) based on the previously published protocol (Rai et al. 2021a, 2021b). The organoids were used at day 95 from the start of the differentiation protocol, and cortical and noncortical markers were assessed to ensure proper differentiation (Rai et al. 2021a).

### MG132 treatment

MG132 (Sigma #M8699, ≥98% purity) dissolved in DMSO (Sigma #472301, ≥99.9% purity) was administered for the indicated treatment durations and doses in culture medium with 10% FBS. Results were compared with DMSO-matched control samples. Specifically, samples exposed to different amounts and durations of DMSO treatments were utilized as controls for the matched samples that were treated with MG132 dissolved in the same amount of DMSO and exposed to the same length of treatment.

There are several considerations related to MG132 treatments. First, MG132 is a widely used proteasome inhibitor, but it also inhibits lysosomal proteases and calpains (Kisselev and Goldberg 2001; Goldberg and Rock 2002; Obeng et al. 2006; Goldberg 2012; Kamens et al. 2023). Therefore, some of the gene expression changes that are observed in response to MG132 treatment may derive from the repression of other proteolytic systems. However, we find that some of the same gene categories are similarly regulated by the siRNA-mediated knockdown of core proteasome components (a strategy previously used to target specifically the proteasome [Lundgren et al. 2005; Tsvetkov et al. 2015; Wang et al. 2017; Lv et al. 2020; Rai et al. 2021a]), suggesting that many of the transcriptional changes observed in response to MG132 treatment indeed derive from proteasome inhibition.

Second, high doses and prolonged treatment with MG132 can induce cell death, and this strategy is utilized for the chemotherapeutic treatment of several cancers (Kisselev and Goldberg 2001; Goldberg and Rock 2002; Obeng et al. 2006; Goldberg 2012; Kamens et al. 2023). Therefore, some cell death occurs in cultured cells treated with MG132. However, the treatment with MG132 was done in the presence of serum which sustains viability in MG132-treated cells. Moreover, apoptotic cells are less adherent and are washed away when removing the culture medium. Consistently, cell death, apoptosis, and related terms are not detected as enriched categories in any of the RNA-seq datasets analyzed here (Figs. 1 to 3), confirming that the transcriptional changes here reported do not come from dead cells.

**Fig. 1.**
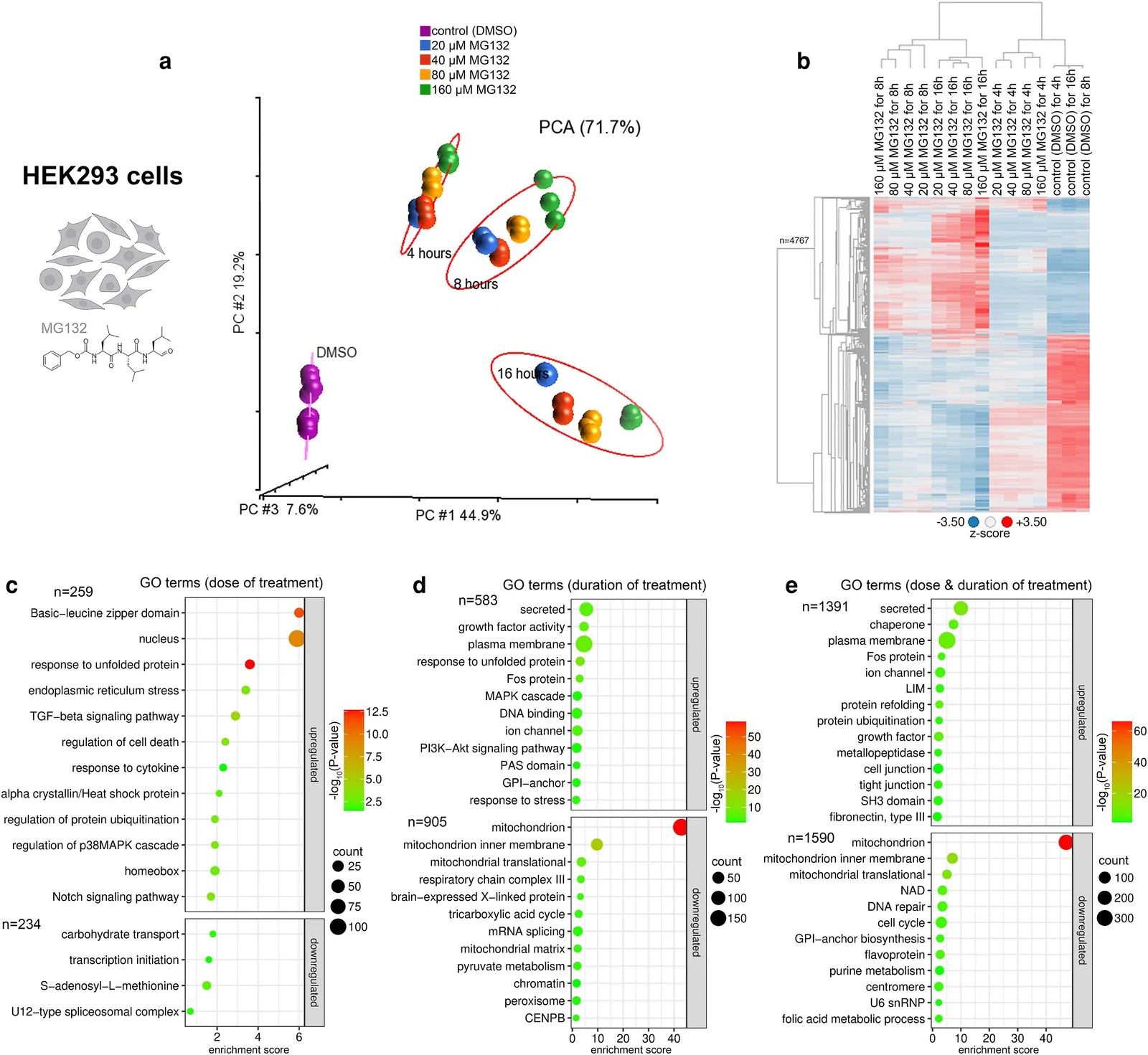
Transcriptional responses induced by proteasome inhibition with different doses and lengths of MG132 treatment in HEK293T cells. Human HEK293T cells were treated with different concentrations of the proteasome inhibitor MG132 (20, 40, 80, 160 µM) compared to the vehicle control (DMSO) for 4, 8, or 16 h (*n* = 3 biological replicates/group). a) PCA indicates a separation of the samples primarily based on the length of treatment and less so based on the MG132 concentration. b) Heatmap of the 4,767 genes that are most highly regulated by MG132 treatment (log_2_  *R* > |1| and *P* < 0.05 in at least one comparison). The *z*-scores were used to visualize upregulated (red) and downregulated (blue) genes but not to determine differential expressions. The most striking changes are found for the longest length (16 h) of treatment rather than for the highest dose (160 µM), although the most prominent transcriptional changes are obtained with the 160 µM treatment for 16 h with MG132. c to e) GO categories that are most prominently modulated among significantly upregulated (*P* < 0.05 and log_2_  *R* > 1) and downregulated (*P* < 0.05 and log_2_  *R* < −1) genes by c) the maximal dose rather than length of treatment (ie 4 h treatment with 160 µM MG132), d) the maximal length rather than dose of treatment (ie 16 h treatment with 20 µM MG132), and e) the highest dose and length of treatment (160 µM MG132 for 16 h). The number of modulated genes, enrichment scores for each category, and −log_10_(*P*-value) are indicated. See also Supplementary Dataset 1. The schemes were created in BioRender (Demontis F., 2026, https://BioRender.com/mxbof8d).

**Fig. 2.**
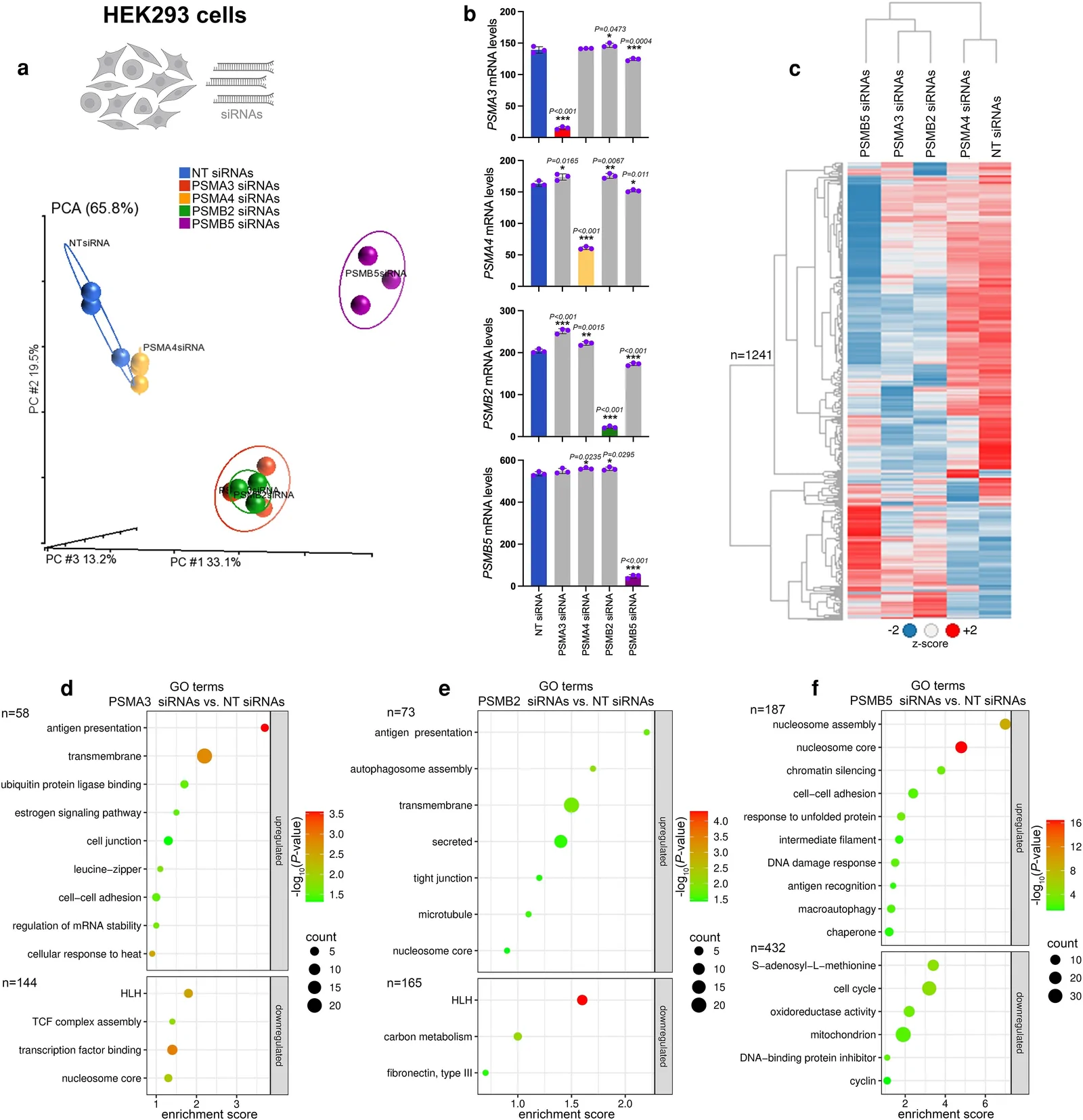
Transcriptional responses induced by genetic inhibition of the proteasome in HEK293T cells. a) Human HEK293T cells were treated with siRNAs targeting components of the catalytic core of the proteasome (PSMA3, PSMA4, PSMB2, and PSMB5) compared to control NT siRNAs. PCA indicates that PSMB5 knockdown (purple) induces the strongest transcriptional response, that siRNAs for PSMB2 (green) and PSMA3 (red) induce overlapping gene expression changes, and that PSMA4 siRNAs (yellow) induce negligible changes compared to control NT siRNAs (*n* = 3 biological replicates/group). b) Treatment with siRNAs significantly reduces the mRNA levels (TPM values) of the intended target genes, with minor but significant adjustments in the levels of other proteasomal components. The graphs display the mean ± SD with *n* = 3 biological replicates/group. The statistical analyses refer to the comparison of each sample to the control NT siRNA sample by 1-way ANOVA (*P < 0.05, **P < 0.01, ***P < 0.001). c) Heatmap of the most-highly regulated genes (*n* = 1,241; *P* < 0.05 and log_2_  *R* > |0.585|, ie >50% change, in at least one comparison) indicates that knockdown of PSMB5 and, secondarily, of PSMA3 and PSMB2 induces the most striking gene expression changes compared to control NT siRNAs, whereas PSMA4 knockdown causes marginal changes. The *z*-scores were used to visualize upregulated (red) and downregulated (blue) genes but not to determine differential expressions. d to f) GO terms enriched in significantly upregulated (*P* < 0.05 and log_2_  *R* > 0.585, ie a 50% increase) and downregulated (*P* < 0.05 and log_2_  *R* < −0.585, ie a 50% decline) genes in cells treated with siRNAs for PSMA3, PSMB2, and PSMB5. The number of modulated genes, enrichment scores for each category, and −log_10_(*P*-value) are indicated. See also Supplementary Dataset 1. https://BioRender.com/mxbof8d

**Fig. 3.**
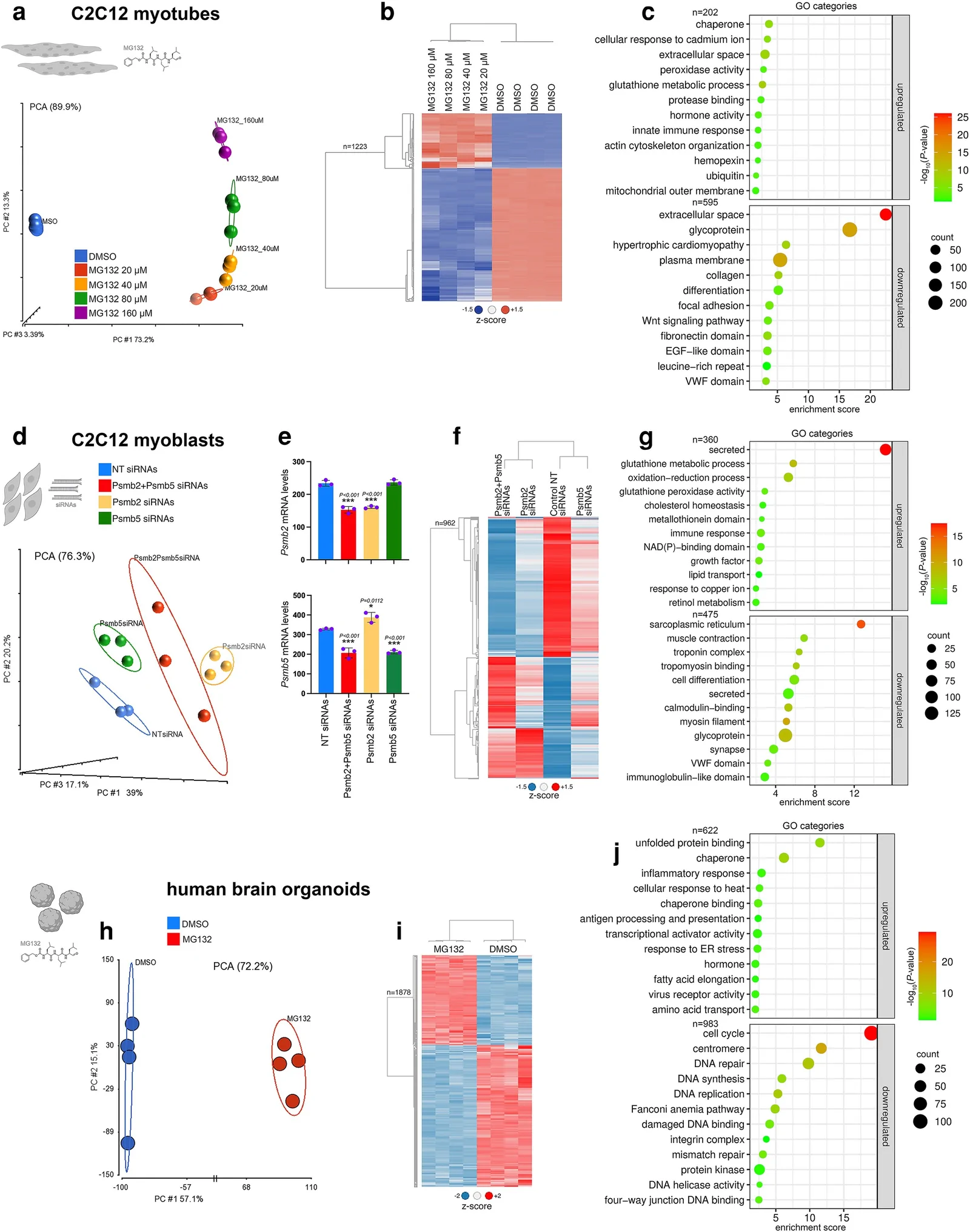
Transcriptional responses induced by proteasome inhibition in C2C12 cells and in human brain organoids. a and b) Mouse C2C12 myotubes were treated with different concentrations of the proteasome inhibitor MG132 (20, 40, 80, 160 µM) compared to corresponding levels of the vehicle alone (DMSO) for 16 h (*n* = 3 biological replicates/group). The PCA a) and heatmap of the most regulated genes (*n* = 1,223; *P* < 0.05 and log_2_  *R* > |2| in at least one comparison) b) indicate that 20 µM MG132 induces profound gene expression changes, and further minor changes are triggered by increasingly higher levels of MG132. c) GO terms enriched in significantly upregulated (*P* < 0.05 and log_2_  *R* > 2) and downregulated (*P* < 0.05 and log_2_  *R* < −2) genes in cells treated with 20 µM MG132 for 16 h. The number of modulated genes, enrichment scores for each category, and −log_10_(*P*-value) are indicated. See also Supplementary Dataset 2. d) PCA plot of the transcriptional changes induced by knockdown of core proteasome components (Psmb2 and Psmb5) in mouse C2C12 myoblasts (*n* = 3 biological replicates/group). e) Treatment with siRNAs for Psmb2 and Psmb5 was done individually and in combination, and resulted in consistent decline in the mRNA levels (TPM values) of the intended target genes. The graphs display the mean ± SD with *n* = 3 biological replicates/group. The statistical analyses refer to the comparison of each sample to the control NT siRNA sample by 1-way ANOVA (*P < 0.05, ***P < 0.001). f) Heatmap of the most-highly regulated genes (*n* = 962; *P* < 0.05 and log_2_  *R* > |0.585|, ie >50% change, in at least one comparison) indicates that the combination of siRNAs for Psmb2 and Psmb5 elicits the most profound changes compared to control treatment with NT siRNAs. g) GO categories enriched among the genes that are upregulated (*P* < 0.05 and log_2_  *R* > 0.585, ie a 50% increase) and downregulated (*P* < 0.05 and log_2_  *R* < −0.585, ie a 50% decline) by Psmb2 + Psmb5 siRNAs vs NT siRNAs. The number of modulated genes, enrichment scores for each category, and −log_10_(*P*-value) are indicated. See also Supplementary Dataset 3. h and i) Human brain (cortical) organoids were treated at day 95 of differentiation with 160 µM MG132 for 8 h compared to treatment with DMSO alone. The PCA h) and heatmap i) of 1,878 highly regulated genes (*P* < 0.05 and log_2_  *R* > |1| in at least one comparison) indicate profound gene expression remodeling in response to proteasome inhibition (*n* = 4 biological replicates/group). j) GO terms enriched among significantly upregulated (*P* < 0.05 and log_2_  *R* > 1) and downregulated (*P* < 0.05 and log_2_  *R* < −1) genes are shown alongside the number of modulated genes, enrichment scores for each category, and −log_10_(*P*-value). See also Supplementary Datasets 4 and 7. https://BioRender.com/mxbof8d.

Third, comparison of the proteasome stress response across different cell culture models indicates that the shared enrichment of common gene categories (Figs. 1 to 3) and upregulation of proteasome components (Fig. 4 and Supplementary Fig. 1) occur in a context-dependent manner (ie depending on the mode of proteasome inhibition and cell type considered). Specifically, the overall and proteasome gene expression changes induced by knockdown of proteasome components are weaker than those obtained with MG132, indicating that the siRNA-mediated knockdown of proteasome components elicits a milder perturbation of the proteasome compared to MG132 treatment.

**Fig. 4.**
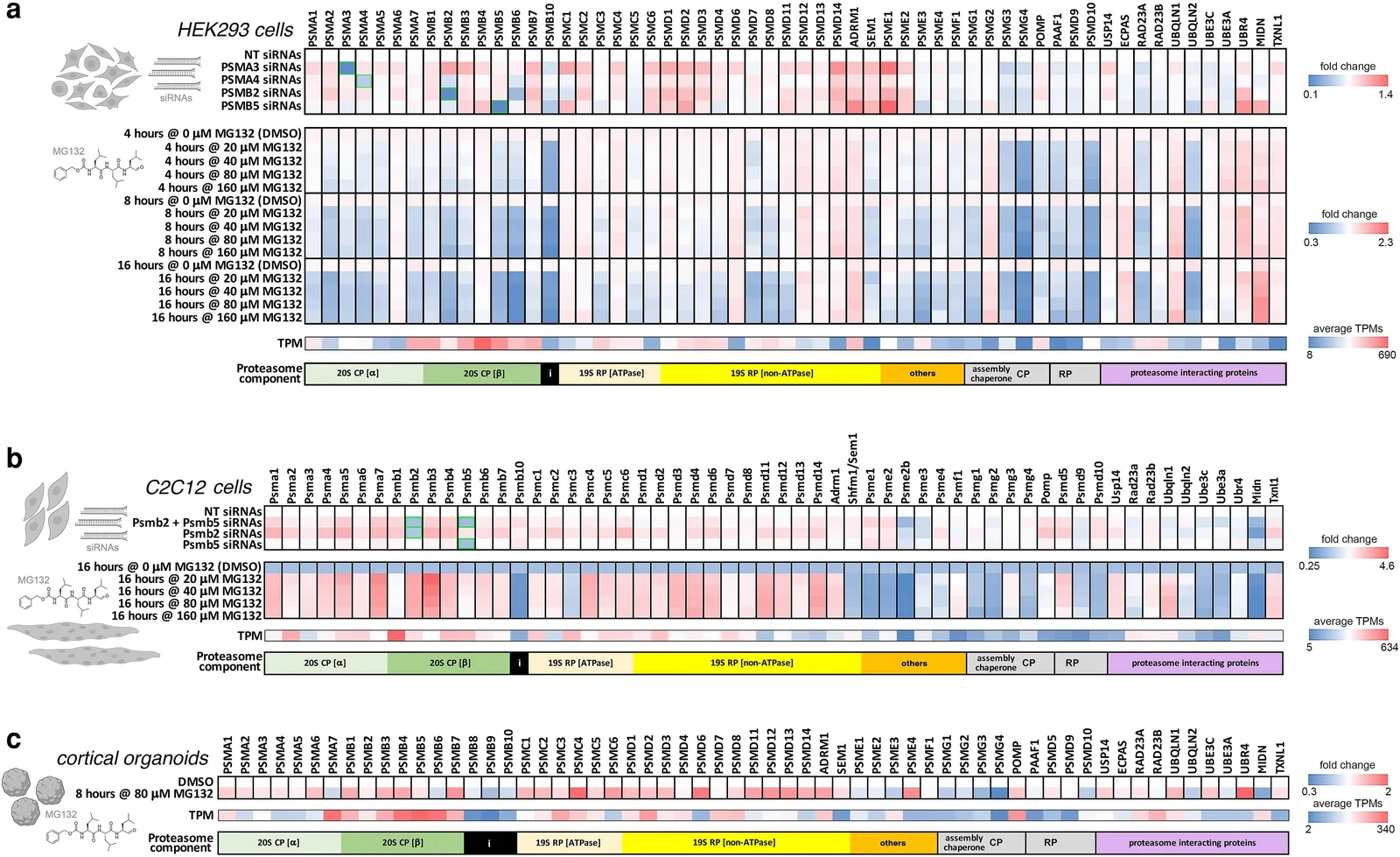
Overview of the changes in proteasome gene expression caused by the chemical and/or genetic inhibition of the proteasome. a to c) Proteasome gene expression in HEK293T cells a), C2C12 myoblasts and myotubes b), and human brain organoids c) in response to inhibition of the proteasome via MG132 a to c) or via siRNA-mediated knockdown of components of the 20S proteasome a and b). The relative fold-change in the expression of each proteasomal component was obtained from *n* = 3 (HEK293T and C2C12 cells) and *n* = 4 (organoids) biological replicates per condition. For each dataset, a color-coding corresponding to the relative fold-change in the expression of each proteasome component is shown across the different conditions (eg MG132 vs DMSO control). In addition, for each cell type, a lower panel displays the average mRNA levels (TPM values) for each proteasome component in control conditions (DMSO). In parallel with a wide range in the expression of distinct proteasome components, proteasome inhibition (via MG132 or knockdown of proteasome subunits) triggers diverse outcomes on proteasome gene expression that vary depending on the proteasome component and cell system. In a to c), the panels display the modulation of proteasome components of the 20S catalytic core (α and β subunits), immunoproteasome subunits (“i;” PSMB8-10), ATPase and non-ATPase components of the 19S RP, other components (eg PSME subunits), assembly chaperones of the CP and RP, and proteasome interacting proteins. The schemes were created in BioRender (Demontis F., 2026, https://BioRender.com/mxbof8d).

### RNA sequencing, hierarchical clustering, and associated analyses

RNA-seq was done as previously reported (Robles-Murguia et al. 2019; Robles-Murguia et al. 2020; Graca et al. 2021; Graca et al. 2023; Hunt et al. 2025a; Curley et al. 2026). RNA-seq libraries were generated from 1 μg of total RNA for each sample using the Illumina TruSeq RNA Sample Prep v2 kit, following the manufacturer's protocol. Sequencing was carried out on an Illumina HiSeq 2000 platform. The resulting 100-bp paired-end reads underwent trimming and quality control, removing sequences with a Phred score below Q20 and discarding reads shorter than 50 bp. Cleaned reads were then aligned to the human reference genome GRCh38 (UCSC hg38) and to the mouse reference genome (GRCm38/mm38) using CLC Genomics Workbench v12.0.1 (Qiagen). Transcript abundance was quantified as transcripts per million (TPM) through the CLC RNA-Seq Analysis tool. Genes with TPM values >1 in at least one dataset were retained for downstream analyses, including principal component analysis (PCA). Differential expression analysis was done using nonparametric ANOVA with Kruskal–Wallis and Dunn's tests on log-transformed TPM values (after adding a pseudocount of 1), implemented in Partek Genomics Suite v7.0 (Partek Inc.). Gene Ontology (GO) term analysis was done using DAVID (https://davidbioinformatics.nih.gov/tools.jsp), and the corresponding graphs were plotted by utilizing Srplot (https://www.bioinformatics.com.cn/srplot) (Tang et al. 2023). The 116 RNA-seq datasets reported in this study were submitted to the Gene Expression Omnibus (GEO) and are accessible at the GEO with the identifier GSE312868.

Protein-coding genes showing significant differential expression between experimental groups (defined as indicated) relative to their matched controls were subjected to hierarchical clustering. Heatmaps were generated using the UPGMA algorithm (unweighted pair-group method with arithmetic mean) with correlation-based similarity metrics. For each gene, *z*-scores were then calculated based on these group-average expression values and used solely for visualization purposes. All clustering and visualization steps were performed using the Spotfire software (Tibco), version 7.5.0.

The Tabula Muris Senis (https://twc-stanford.shinyapps.io/maca/) (Tabula Muris 2020) was consulted to define the trajectory of gene expression during aging of the genes that are upregulated and downregulated by proteasome stress across multiple cell types (proteasome stress signature). The aging plasma proteome database (https://twc-stanford.shinyapps.io/aging_plasma_proteome/) (Lehallier et al. 2019) was consulted to examine how secreted factors induced by proteasome stress are correspondingly modulated by aging in the human plasma. The harmonizome website (Rouillard et al. 2016; Diamant et al. 2025) was consulted to retrieve ChIP-seq data from the ENCODE Transcription Factor Targets database.

### Statistical analysis

Data organization and graphing were done with Microsoft Excel (version 14.7.3), GraphPad Prism (version 10), and Srplot (https://www.bioinformatics.com.cn/srplot). The test utilized for statistical analysis is indicated in the figure legends. The bar graphs display the mean ± SD, as indicated in the legends. A significant result was defined as *P* < 0.05. Throughout the figures, asterisks indicate the significance of *P* values: *P < 0.05, **P < 0.01, and ***P < 0.001.

## Results

### Time–dose effects of chemical perturbation of the proteasome

The reversible, cell-permeable proteasome inhibitor MG132 (carbobenzoxy-Leu-Leu-leucinal) is one of the tools that is most frequently utilized to perturb proteasome function in experimental settings (Bush et al. 1997; Lee and Goldberg 1998; Goldberg 2012; Sha and Goldberg 2014). This inhibitor is related to bortezomib, which is the standard of care for multiple myeloma and is increasingly considered as a viable chemotherapeutic option for other cancer types (Kisselev and Goldberg 2001; Goldberg and Rock 2002; Obeng et al. 2006; Goldberg 2012; Kamens et al. 2023). To determine the transcriptional changes induced by MG132, human HEK293T cells were treated with different concentrations of MG132 (dissolved in DMSO) for different periods compared to mock treatments (ie same amounts of the DMSO vehicle), and the RNA was prepared and analyzed by RNA-seq. PCA and the heatmap of most regulated genes (*P* < 0.05 and log_2_  *R* > |1| in at least one comparison) indicate that there are bigger changes following a longer duration of treatment rather than in response to higher doses of MG132. In particular, a greater divergence was observed between the samples treated for 16 h vs those treated for 8 and 4 h and the untreated (DMSO) controls, compared to the transcriptional differences found in the samples treated with 160 µM MG132 vs those treated with 80, 40, and 20 µM MG132 and the untreated controls (Fig. 1, a and b). A similar conclusion is also reached from the analysis of the GO terms that are enriched among the significantly regulated genes: both the number of genes and the enrichment scores of modulated categories are overall lower for the dose (ie 160 µM for 4 h; Fig. 1c) vs the treatment duration (ie 20 µM for 16 h; Fig. 1d). Analyses of the transcriptional changes triggered by maximal time–dose treatments (ie 160 µM for 16 h) indicate that MG132-induced categories include secreted factors, chaperones/response to unfolded proteins, plasma membrane proteins, ion channels, regulators of ubiquitination, and cell adhesion proteins, whereas MG132-downregulated genes encode for mitochondrial proteins and cell cycle regulators (Fig. 1e). As explained above, most of these responses are attributed to longer duration of treatment with MG132 rather than from a higher dose. However, chaperones and related gene categories are commonly induced by MG132 irrespective of time–dose differences. Altogether, these gene expression analyses provide insight into the progressive changes that occur in response to different concentrations and durations of MG132 treatment.

### Transcriptional changes induced by genetic perturbation of the proteasome

In addition to the chemical perturbation of proteasome function with MG132, previous studies found that knockdown of proteasome components can be utilized to moderately impair proteasome activity (Belote and Fortier 2002; Wojcik and DeMartino 2002; Lundgren et al. 2005; Rai et al. 2021a). To this purpose, we next utilized siRNAs to target specific components (PSMA3, PSMA4, PSMB2, and PSMB5) of the catalytic core of the proteasome (ie the 20S proteasome composed of α and β subunits). These proteasome components are key for proteasome activity and were previously targeted in other studies to induce the proteasome stress response and/or reduce proteasome activity (Lundgren et al. 2005; Tsvetkov et al. 2015; Wang et al. 2017; Lv et al. 2020; Rai et al. 2021a).

Compared to control NT siRNAs, treatment of HEK293T cells with these siRNAs followed by RNA-seq analyses revealed that the highest responses are induced by PSMB5 siRNAs followed by PSMA3 and PSMB2 siRNAs, whereas PSMA4 siRNAs had minimal effects (Fig. 2a). Supporting these observations, analysis of the mRNA levels (TPM values) for the siRNA-targeted genes indicates that siRNAs against PSMB5, PSMB2 and PSMA3 achieved the strongest knockdown (∼90%), whereas PSMA4 siRNAs led to a significant but weaker (∼60%) knockdown (Fig. 2b). Interestingly, while these siRNA-based interventions specifically targeted the intended proteasome component, there were small but significant changes in some of the other monitored proteasome proteins (Fig. 2b). However, these adjustments did not occur univocally, ie in some cases knockdown of a proteasome component triggered the upregulation of other components, but sometimes the converse occurred. For example, PSMA3 knockdown mildly reduced PSMB5 mRNA levels, whereas PSMB2 knockdown siRNAs increased PSMA3 mRNA levels (Fig. 2b). Heatmap analysis of the most highly modulated genes (*P* < 0.05 and log_2_  *R* > |0.585|, ie >50% change, in at least one comparison) further demonstrated the differential efficacy of siRNAs targeting distinct proteasome components, with PSMB5 knockdown delivering the highest changes (Fig. 2c). The categories that were commonly upregulated by the strongest interventions (PSMB5, PSMB2, and PSMA3 knockdown) included antigen presentation/recognition, cell adhesion, chaperones, and autophagy, whereas helix-loop-helix (HLH)/DNA-binding proteins and cell cycle regulators were among the downregulated terms (Fig. 2d to f). Compared to the chemical inhibition of the proteasome with MG132 (Fig. 1), knockdown of proteasome components induced fewer changes, but some categories were commonly regulated (eg chaperones and cell adhesion). Altogether, these analyses define the transcriptional changes induced by genetic perturbation of α and β subunits of the catalytic core of the proteasome in human cells.

### The proteasome stress response in myotubes, myoblasts, and human brain organoids

The comprehensive analyses reported above define the stress responses induced by the chemical (Fig. 1) and genetic (Fig. 2) perturbations of the proteasome in HEK293T cells, a widely used human cell line. As a result, the datasets generated here contribute to the growing resources that are available to investigate proteostasis and stress responses in this cell line. However, the proteasome stress response may occur differently in other cell types and models. To address this issue, we subsequently utilized mouse C2C12 myotubes, C2C12 myoblasts, and human brain (cortical) organoids to determine the transcriptional changes induced by proteasome perturbation. C2C12 myotubes were treated with increasing doses of MG132 (20, 40, 80, 160 µM) for 16 h and compared to equal amounts of DMSO as mock treatments. Both the PCA (Fig. 3a) and the heatmap of the 1,223 (*P* < 0.05 and log_2_  *R* > |2| in at least one comparison) most regulated genes (Fig. 3b) indicate that similar transcriptional responses are induced by different doses of MG132. Genes that are transcriptionally induced by MG132 are enriched for chaperones, extracellular proteins, and peroxidases. Extracellular proteins, glycoproteins, and plasma membrane proteins are overrepresented among downregulated proteins (Fig. 3c).

Due to the greater efficiency of siRNA transfection observed in myoblasts compared to myotubes (Graca et al. 2022; Stephan et al. 2022), we utilized mouse C2C12 myoblasts for the siRNA-mediated knockdown of β subunits of the 20S proteasome, ie Psmb2 and Psmb5 (Fig. 3d). A similar reduction in Psmb2 and Psmb5 mRNA levels was obtained by both the individual and combined knockdown of these subunits (Fig. 3e). Clustering analysis of the most highly regulated genes indicates that the combination of siRNAs targeting Psmb2 and Psmb5 induces the highest transcriptional changes (Fig. 3f). Upregulated gene categories include secreted factors, glutathione metabolism, cholesterol homeostasis, and immune responses, whereas genes encoding for contractile proteins are downregulated (Fig. 3g).

Human brain organoids have emerged as useful experimental models (Hu et al. 2018). Organoids can be differentiated following protocols tailored to different brain areas, such as the neocortex (Qian et al. 2016). A previous study utilized brain (cortical) organoids to examine proteostasis and the outcome of systemic proteasome stress signaling (Rai et al. 2021a, 2021b). Here, we have treated brain (cortical) organoids (differentiated through the same protocol) with 160 µM MG132 for 8 h, enabling the characterization of the transcriptional response to proteasome inhibition in this model (Fig. 3, h and i). Upregulated categories include chaperones, inflammatory mediators, and antigen processing/presentation, whereas downregulated categories comprise the cell cycle and DNA replication (Fig. 3j). Altogether, these analyses define the gene expression changes induced by proteasome stress in myotubes, myoblasts, and brain organoids.

### Inhibition of the proteasome induces context-dependent changes in the expression of proteasome components

Previous studies reported that a key outcome of proteasome inhibition is the induction of proteasome gene expression and the reestablishment of proteasome activity via the assembly of new proteasomes (Rai et al. 2025b). This transcriptional response was found to depend on proteasome-sensitive transcription factors that are rapidly degraded when the proteasome is functional but that are instead stabilized in response to proteasome inhibition (ie Rpn4 in yeast and Nrf1/Nfe2l1 in higher organisms) (Xie and Varshavsky 2001; Lundgren et al. 2005; Steffen et al. 2010; Radhakrishnan et al. 2014; Sha and Goldberg 2014; Koizumi et al. 2016; Lehrbach and Ruvkun 2016; Lehrbach et al. 2019; Dirac-Svejstrup et al. 2020). On this basis, we examined the modulation of proteasome gene expression in HEK293 cells and C2C12 myoblasts treated with siRNAs for 20S proteasome components (Fig. 4, a and b), and in HEK293 cells (Fig. 4a and Supplementary Fig. 1), C2C12 myotubes (Fig. 4b) and brain organoids treated with MG132 (Fig. 4c).

In these datasets, there was increased expression of many proteasome genes, including those that encode components of the 20S catalytic core (α and β subunits), ATPase and non-ATPase components of the 19S regulatory particle (RP), and (to a lower extent) also for other components (eg PSME, proteasome activator subunits), assembly chaperones of the core particle (CP) and RP, and proteasome interacting proteins (Fig. 4), such as the E3 ubiquitin ligase UBR4 which physically binds to the proteasome (Besche et al. 2009; Hunt et al. 2019b; Hunt et al. 2021). However, subunits of the immunoproteasome (PSMB8, PSMB9, and PMSB10) were repressed (Fig. 4).

Interestingly, the regulation of each specific proteasome component varied depending on the cell type, genetic vs chemical proteasome inhibition, and the time and dose of treatment (Fig. 4a to c). Altogether, these analyses indicate that proteasome gene expression is upregulated to varying degrees and in a context-dependent manner in response to proteasome inhibition (Fig. 4a to c). However, while the expression of proteasome components generally increases (Fig. 4), it appears that this is not the most prominent and widespread gene category that is induced by proteasome inhibition across different cell types (Figs. 1 to 3).

### Definition of a transcriptional signature of proteasome stress

We next defined a conserved signature of proteasome stress by examining the conserved transcriptional changes triggered by proteasome inhibition in HEK293T cells, C2C12 myotubes, and human brain organoids. Because MG132 induced the strongest responses compared to the siRNA-mediated knockdown of individual proteasome components (Figs. 1 to 3), we utilized the maximal time–dose responses induced by MG132 in HEK293T cells, C2C12 myotubes, and brain organoids to identify a defined set of genes that are concordantly and significantly regulated and with at least a 2-fold change across all these datasets. Through this approach, we identified 67 genes that are upregulated and 54 genes that are downregulated in response to MG132 treatment (Fig. 5a). This set of 121 genes was enriched for chaperones, secreted proteins, transcription factors, and cell cycle regulators (Fig. 5b).

**Fig. 5.**
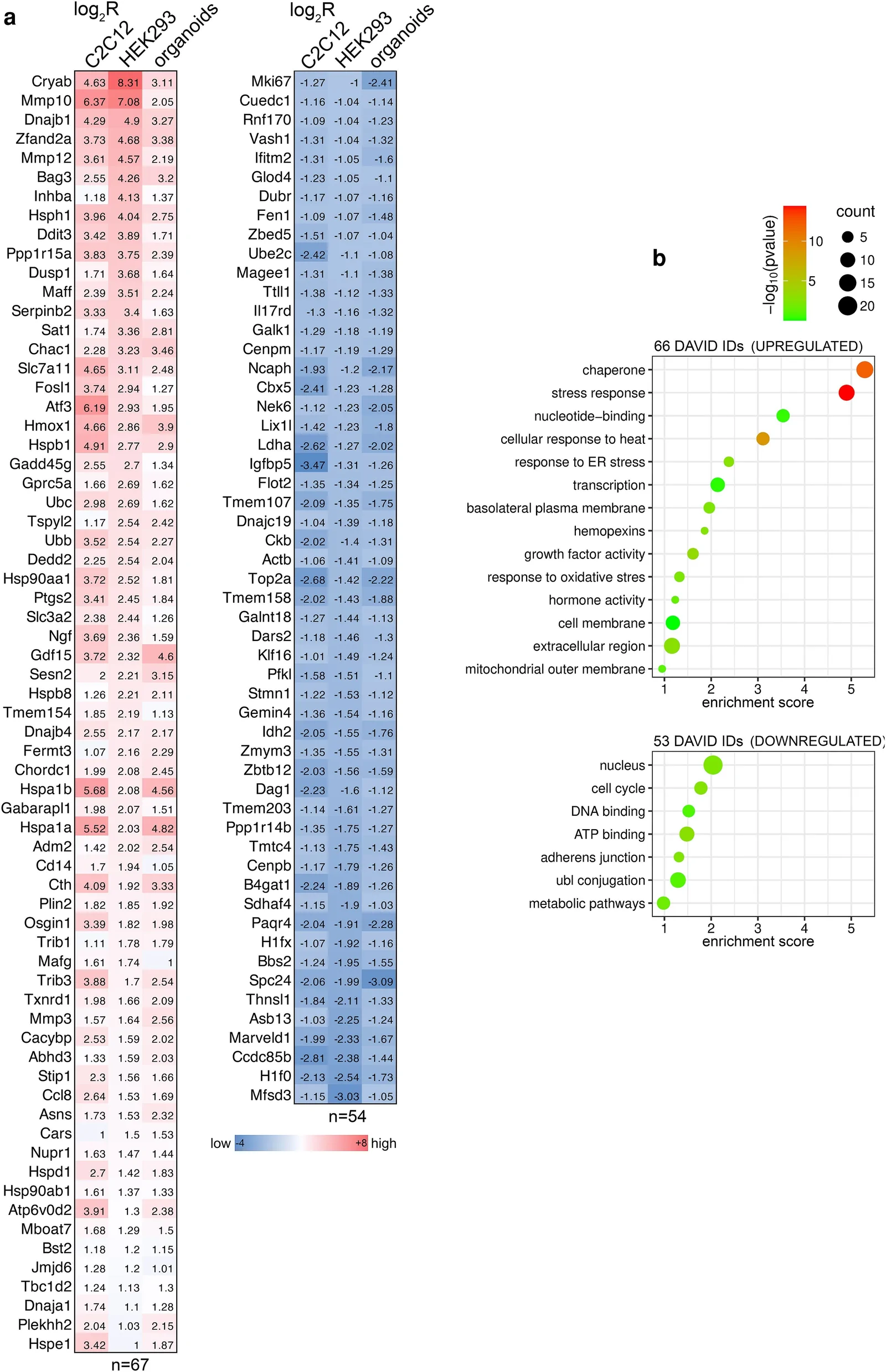
A conserved transcriptional signature of proteasome stress. a) Cross-comparison of the gene expression changes induced by MG132 in HEK293T cells (160 µM MG132 for 16 h), C2C12 myotubes (160 µM MG132 for 16 h), and human brain organoids (160 µM MG132 for 8 h). The genes that are consistently upregulated (log_2_  *R* > 1 and *P* < 0.05; *n* = 67) and downregulated (log_2_  *R* < −1 and *P* < 0.05; *n* = 54) across all 3 cell systems were selected because they are cross-shared readouts of proteasome inhibition (“proteasome stress signature;” *n* = 121). b) GO term analysis of the signature genes. Categories that are enriched among the stress-upregulated signature genes include chaperones, regulators of stress responses, transcription, and secreted proteins (eg growth factors and hormones), whereas nuclear proteins and regulators of the cell cycle, cell adhesion, and ubiquitination are overrepresented among the stress-downregulated signature genes. The number of modulated genes, enrichment scores for each category, and −log_10_(*P*-value) are indicated.

Previous studies uncovered several specialized unfolded protein responses induced by perturbation of different organelles and cell compartments, such as the UPR^ER^ and UPR^mt^ (Marciniak and Ron 2006; Kourtis and Tavernarakis 2011). For example, the mitochondrial UPR^mt^ triggers the upregulation of mitochondrial chaperones (eg Hsp60) to restore mitochondrial proteostasis (Jovaisaite et al. 2014; Munch 2018). Interestingly, induction of chaperones is a prominent, common feature of the transcriptional response to proteasome inhibition (Figs. 1 to 3), as reported in earlier studies (Bush et al. 1997; Lee and Goldberg 1998). The proteasome stress signature defined here also includes several heat shock proteins, such as Cryab, Bag3, Hsph1, Hspb1, Hsp90aa1, and Hspb8 (Fig. 5a). These chaperones belong to diverse families and are located in different subcellular compartments but are found primarily in the nucleus and cytoplasm, suggesting that the proteasome stress response is similar to a cytoplasmic unfolded protein response. However, comparison of the proteasome stress response signature to the transcriptional changes induced by a cytoplasmic misfolding-prone protein (Fluc^DM^ [Curley et al. 2024]) expressed in HEK293T cells indicates minimal similarity (Supplementary Fig. 2), suggesting that different unfolded protein response (UPR) variants are induced by these distinct triggers.

Among the signature genes, Cryab (crystallin alpha B) emerges as the top-induced chaperone by MG132 in HEK293T cells, C2C12 myotubes, and human brain organoids (Fig. 5a). The Drosophila homolog of this small heat shock protein, Hsp23, was similarly identified as the top-upregulated chaperone in response to proteasome stress in Drosophila (Rai et al. 2021a). Interestingly, also Ubb and Ubc (which encode for ubiquitin) are also cross-induced by proteasome stress (Fig. 5a), suggesting that ubiquitin induction is a component of proteasome inhibition, presumably because of decreased proteasome-mediated recycling of ubiquitin and consequent depletion of free ubiquitin, a condition known as ubiquitin stress which triggers ubiquitin gene expression (Rai et al. 2025b). This occurs concomitantly to a decline in Ube2c (Fig. 5a), an E2 ubiquitin-conjugating enzyme, and this adaptation may help conserve sufficient levels of free ubiquitin to ensure cell homeostasis. Moreover, also the Atg8/LC3-like protein Gabarapl1 and regulators of lipid metabolism (Plin2 and Abhd3) are induced by proteasome stress and are part of the proteasome stress signature (Fig. 5a). Collectively, several gene categories, including chaperones, are overrepresented in the signature of proteasome stress.

### Potential transcription factors mediating the proteasome stress response

Nrf1/Nfe2l1 is the primary transcription factor that was reported to mediate the transcriptional response to proteasome stress (Radhakrishnan et al. 2010; Radhakrishnan et al. 2014; Sha and Goldberg 2014; Koizumi et al. 2016; Lehrbach and Ruvkun 2016; Lehrbach et al. 2019; Dirac-Svejstrup et al. 2020; Rai et al. 2025b).

Nrf1/Nfe2l1 binds DNA as a heterodimer with Maf transcription factors (eg Maff and Mafg) (Johnsen et al. 1998), which are transcriptionally induced by proteasome stress and are part of the proteasome stress signature (Fig. 5a). On this basis, we consulted ChIP-seq data from the ENCODE Transcription Factor Targets dataset through the Harmonizome website (Rouillard et al. 2016; Diamant et al. 2025), which includes 9,321 previously reported Maff binding sites, to determine whether Maff binding is enriched at the promoters of the genes that are part of the proteasome stress signature and that are consistently upregulated (*n* = 67) and downregulated (*n* = 54) by proteasome stress (Fig. 5a). These analyses revealed that 41/67 upregulated genes and 29/54 downregulated genes display Maff binding sites (Supplementary Dataset 7). Statistical analysis with the χ^2^ test indicates that this is observed significantly more than expected for both upregulated (*P* < 0.01) and downregulated (*P* < 0.001) genes, indicating that Maff transcription factors contribute to the transcriptional response to proteasome stress, presumably as heterodimers with Nrf1/Nfe2l1.

Chaperones are a prominent category of genes that is upregulated by proteasome stress (Figs. 1 to 3). The heat shock factor 1 is known to mediate the response to unfolded proteins and chaperone expression, and therefore, we examined whether any of the 233 binding sites for Hsf1 reported by the ENCODE database is enriched in the genes that are part of the proteasome stress signature (Fig. 5a). This analysis revealed that 18/67 upregulated genes contain a Hsf1 binding site, which is observed significantly more than expected (*P* < 0.001), whereas no downregulated genes carried a Hsf1 binding site, which is not different than statistically expected (Supplementary Dataset 7).

Collectively, these findings indicate that Maff-Nrf1/Nfe2l1 significantly contributes to the induction of signature genes that are upregulated and downregulated by proteasome stress, whereas Hsf1 significantly contributes to induce (but not to repress) gene expression in response to proteasome stress. In addition to Hsf1 and Maff-Nrf1/Nfe2l1, it is also interesting to note that several transcription factors are modulated by proteasome stress at the mRNA level (Fig. 5a), and that therefore may contribute to the ensuing gene expression changes. The upregulated transcription factors include Maff and Mafg (which form heterodimers with Nrf1/Nfe2l1; Johnsen et al. 1998), Fosl1, and Atf3, whereas Zbed5, Klf16, Zmym3, and Zbtb12 are downregulated (Fig. 5a). Collectively, these transcription factors may contribute to the transcriptional responses to proteasome stress.

### Modulation of the proteasome stress signature across aging tissues and organs in mice

We have defined a set of genes (signature) that are recurrently induced by proteasome stress across diverse cell types (Fig. 5a). Similar to other conserved responses induced by other stressors (Murray et al. 2004; Han et al. 2008; Kelley et al. 2025; Wang et al. 2025), we propose that this gene set may represent a transcriptional signature that can be employed to monitor the activation of the proteasome stress response.

As a proof of principle, we examined whether this proteasome stress signature is detected across aging tissues and organs in mice. To this purpose, we consulted the Tabula Muris Senis, a compendium of bulk RNA-seq data that was obtained from different tissues and organs across the mouse lifespan (Tabula Muris 2020). Interrogation of this dataset using the proteasome stress signature indicates that certain aging tissues display a transcriptional modulation of the proteasome stress signature that is consistent with the activation of the proteasome stress response with aging. Specifically, stress-upregulated signature genes show increased expression with aging in the brown fat, gonadal fat, heart, kidney, limb muscle, lung, bone marrow, and spleen. By contrast, stress-downregulated signature genes show reduced expression with aging primarily in the bone, brain, limb muscle, and mesenteric fat. In the limb muscle (ie skeletal muscle), the age-associated increase in stress-upregulated signature gene expression coincides with a decline in stress-downregulated signature gene expression. These changes emerge early in life and are largely maintained throughout aging. A similar collective regulation of the proteasome stress signature (ie both stress-upregulated and stress-downregulated signature genes) is also observed in gonadal fat, heart, kidney, and lung, although with varying fluctuations and to different extents (Fig. 6). Collectively, these analyses suggest that the proteasome stress response is activated during aging in some tissues and organs, presumably in response to perturbation of proteasome function in these tissues by age-associated challenges to proteostasis.

**Fig. 6.**
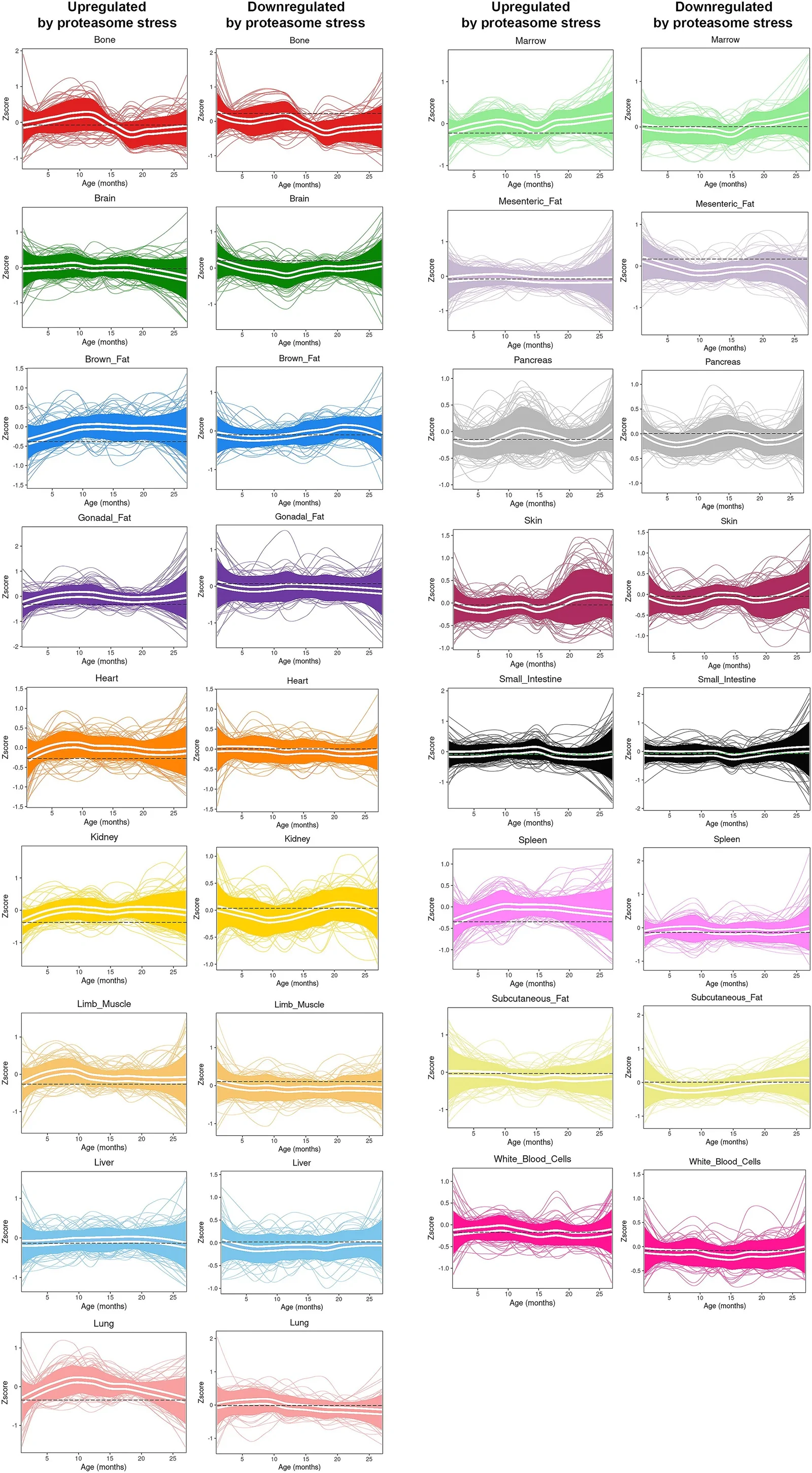
Analysis of the proteasome stress response signature during aging. The Tabula Muris Senis (tabula-muris-senis.sf.czbiohub.org) was consulted to retrieve the collective gene expression trajectories for the 67 stress-upregulated genes that are part of the proteasome stress response signature, and the 54 stress-downregulated signature genes. This analysis indicates that these signature genes are modulated by aging in a manner consistent with the activation of the proteasome stress response in certain tissues and organs. In particular, stress-upregulated signature genes display increased expression with aging in the brown fat, gonadal fat, heart, kidney, limb muscle, lung, bone marrow, and spleen. Stress-downregulated signature genes are less expressed with aging primarily in the bone, brain, limb muscle, and mesenteric fat. The limb muscle (ie skeletal muscle) displays an age-increased expression of proteasome stress-upregulated signature genes that is coincident with the age-associated decline in the expression of proteasome stress-downregulated signature genes. These changes occur early in the lifespan and are then fairly maintained through aging. A similar coincident regulation of the entire proteasome stress signature (ie stress-upregulated and downregulated signature genes) occurs (albeit with varying fluctuations and to different extents) also in the gonadal fat, heart, kidney, and lungs. See also Supplementary Datasets 5 and 7.

### Probing the proteasome stress signature by examining proteomic changes in the human plasma with aging

An unexpected category that is induced by proteasome stress consists of secreted proteins, including signaling factors and components of the extracellular matrix (Figs. 1 to 3 and 5, a and b). While most studies on proteasome stress focused on intracellular mechanisms and did not necessarily highlight the induction of signaling factors that may mediate intercellular signaling (Rai et al. 2025b), there is an extensive literature in this area in the context of aging (Taylor et al. 2014). In particular, previous studies highlighted the induction of systemic stress responses in distant tissues following local stress in a tissue (Demontis and Perrimon 2010; Durieux et al. 2011; Owusu-Ansah et al. 2013; Taylor and Dillin 2013; van Oosten-Hawle et al. 2013; Demontis et al. 2014; Patel and Demontis 2014).

While there are relatively few studies on the proteasome stress response compared to other well-known UPRs, previous studies found that moderate perturbation of proteasome function in skeletal muscle induces local and systemic compensatory responses in Drosophila (Rai et al. 2021a; Rai et al. 2025a). On this basis, we examined how aging regulates the levels of secreted factors that are part of the proteasome stress signature. To this purpose, we consulted the aging plasma proteome database that reports the modulation of circulating factors in the plasma of men and women across the lifespan (Lehallier et al. 2019). This dataset contained data for 6 secreted factors that are part of the proteasome stress signature, and these secreted factors were modulated by aging (Fig. 7) in a manner consistent with their modulation by proteasome stress (Fig. 5a). Specifically, aging and proteasome stress similarly increase the levels of the metalloproteases MMP10 and MMP12, inhibin βA (INHBA), nerve growth factor (NGF), and GDF15 (growth differentiation factor 15, which is known to be induced by other stressors and mediates many organism-wide responses, such as anorexia [Wang et al. 2021]). Conversely, the levels of IL17RD (interleukin 17 receptor D, which can be proteolytically cleaved and released as a soluble form [Girondel and Meloche 2021]) were downregulated with aging (Fig. 7), as also observed in response to proteasome stress (Fig. 5a). Importantly, there was a significant modulation of these factors with aging irrespective of the sex, although there were significant differences for some of these factors when comparing the trajectory of their modulation with aging in men vs women (Fig. 7).

**Fig. 7.**
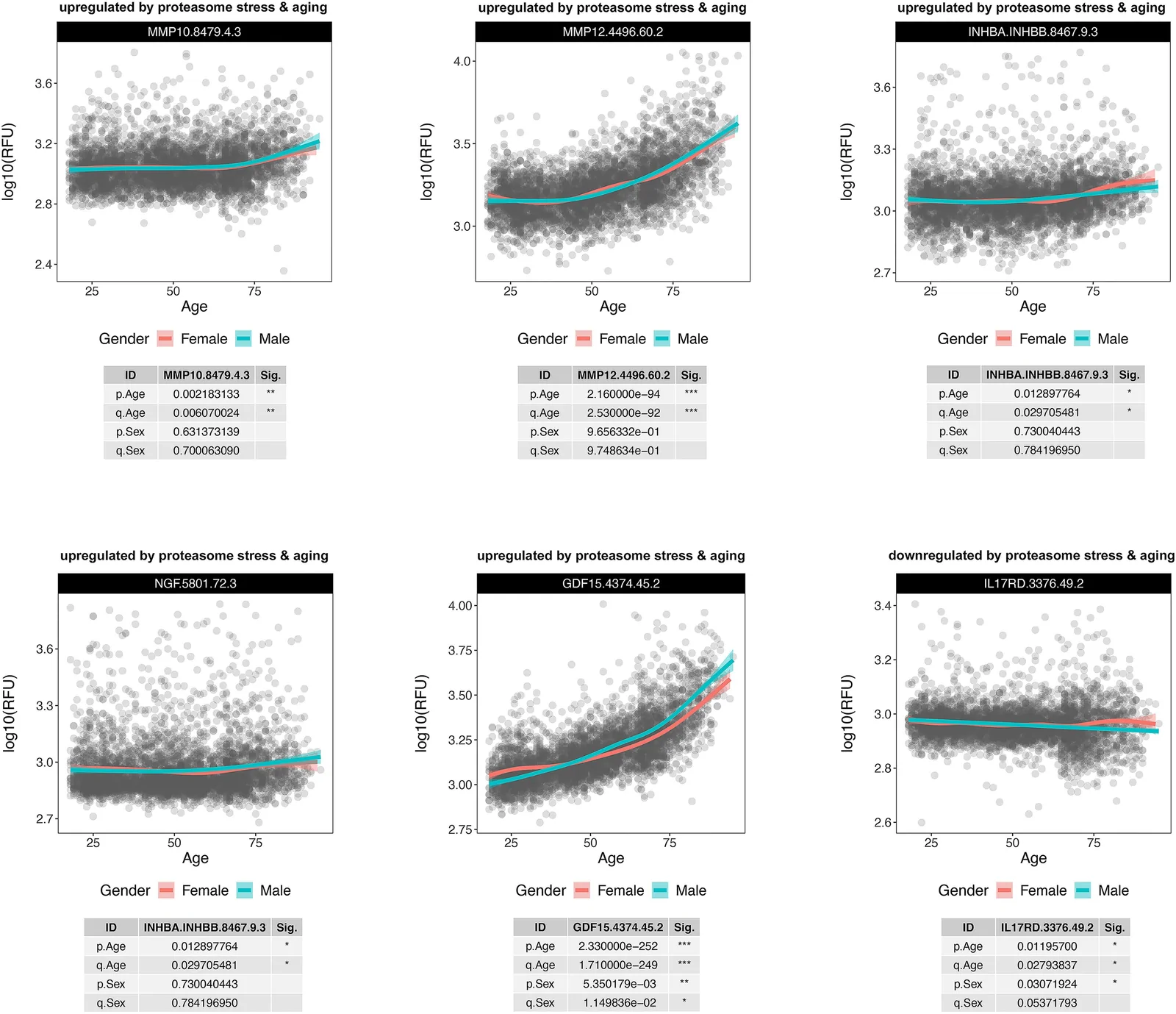
Secreted factors that are part of the proteasome stress response signature are similarly modulated in the human plasma during aging. The aging plasma proteome database (https://twc-stanford.shinyapps.io/aging_plasma_proteome/) was consulted to retrieve information on the secreted factors that are included in the proteasome stress signature. This analysis retrieved information for MMP10, MMP12, INHBA, NGF, and GDF15, which are upregulated by proteasome stress, and IL17RD, which is downregulated. The trajectories of the abundance of these secreted factors over time are indicated for women (pink) and men (blue) alongside the *P*-values and *q*-values for their modulation by age and sex. Collectively, secreted factors that are induced by proteasome stress are also significantly upregulated in the human plasma during aging, whereas IL17RD is downregulated, as observed in response to proteasome stress.

Altogether, secreted signaling factors are a prominent category modulated by proteasome stress across diverse cell types (Fig. 6) and are possible systemic readouts of the proteasome stress response during aging in humans (Fig. 7).

## Discussion

The proteasome maintains cellular homeostasis by degrading damaged or misfolded proteins (Bedford et al. 2011; Schmidt and Finley 2014; Collins and Goldberg 2017), and its perturbation is known to activate backup mechanisms (Rai et al. 2025b). Transcriptional induction of proteasome components is triggered upon proteasome inhibition (Rai et al. 2025b), but it remained undetermined whether this is the primary response induced by proteasome inhibition and whether this occurs equally in different cell types. Here, we addressed this question by comparing MG132- and siRNA-mediated proteasome inhibition in HEK293T cells, brain organoids, and C2C12 myotubes and myoblasts. We find that expression of proteasome components occurred after both genetic (siRNA-mediated) and chemical (MG132-mediated) proteasome inhibition but that this response varied depending on the cell type and mode of proteasome perturbation (Fig. 4). Interestingly, other transcriptional responses were more prominent and shared across different cell systems, including upregulation of chaperones and secreted factors and downregulation of cell cycle regulators (Figs. 1 to 3). Moreover, by identifying the transcriptional responses that are induced by proteasome inhibition across different cell systems, we have defined a transcriptional signature that can be used to probe transcriptional datasets and determine whether the proteasome stress response is modulated in physiological and pathological contexts (Fig. 5). In particular, while the proteasome stress response may influence the outcome of cancer therapies centered on proteasome inhibitors (Kisselev and Goldberg 2001; Goldberg and Rock 2002; Obeng et al. 2006; Goldberg 2012; Kamens et al. 2023), aging may also trigger this stress response because of the many age-associated challenges to proteasome function and proteostasis (Bedford et al. 2011; Schmidt and Finley 2014; Collins and Goldberg 2017; Chocron et al. 2022; Stephan et al. 2026). Consistent with this view, a survey of the Tabula Muris Senis (Tabula Muris 2020) suggests that the proteasome stress response may become activated in some tissues during aging in mice, particularly skeletal muscle (Fig. 6), which is highly vulnerable to age-associated proteostasis decline compared to other tissues (Demontis and Perrimon 2010; Demontis et al. 2013; Jiao and Demontis 2017). Moreover, a survey of the plasma proteomic changes that occur with aging in humans (Lehallier et al. 2019) identified secreted signaling factors that are modulated consistently with their regulation by proteasome stress (Fig. 7), suggesting that aging may induce the proteasome stress response and the ensuing systemic signaling also in humans.

Altogether, this study contributes to defining the transcriptional responses to chemical and genetic inhibition of the proteasome in diverse cell types. We propose that these datasets may provide a useful resource for future studies in this area, and that they will help identify local and systemic stress responses induced by proteasome perturbation. Moreover, the transcriptional signature that we have defined in this study could be used to monitor activation of the proteasome stress response during aging, age-related conditions, and other contexts. However, a limitation of this study is that the signature was established from cells treated with high levels of MG132 because there were a higher variability and a lower magnitude of transcriptional changes in cells with a milder proteasome inhibition (obtained from siRNA-mediated knockdown of proteasome components). Therefore, the proteasome stress signature reported here may be effective in detecting severe proteasome impairment but might be less suitable for monitoring minor perturbations in proteasome activity.

## Supplementary Material

jkag245_Supplementary_Data

## Data Availability

The 116 RNA-seq datasets reported in this study were submitted to the GEO and are accessible at the GEO with the identifier GSE312868. These RNA-seq datasets and additional data analyses are provided in Supplementary Datasets 1 to 7. Supplemental material available at G3 online.

## References

[jkag245-B1] Arndt V et al 2010. Chaperone-assisted selective autophagy is essential for muscle maintenance. Curr Biol. 20:143–148. 10.1016/j.cub.2009.11.022. 20060297

[jkag245-B2] Bedford L, Lowe J, Dick LR, Mayer RJ, Brownell JE. 2011. Ubiquitin-like protein conjugation and the ubiquitin-proteasome system as drug targets. Nat Rev Drug Discov. 10:29–46. 10.1038/nrd3321. PMC7097807 21151032

[jkag245-B3] Belote JM, Fortier E. 2002. Targeted expression of dominant negative proteasome mutants in *Drosophila melanogaster*. Genesis. 34:80–82. 10.1002/gene.10131. 12324954

[jkag245-B4] Besche HC, Haas W, Gygi SP, Goldberg AL. 2009. Isolation of mammalian 26S proteasomes and p97/VCP complexes using the ubiquitin-like domain from HHR23B reveals novel proteasome-associated proteins. Biochemistry. 48:2538–2549. 10.1021/bi802198q. PMC3811022 19182904

[jkag245-B5] Bush KT, Goldberg AL, Nigam SK. 1997. Proteasome inhibition leads to a heat-shock response, induction of endoplasmic reticulum chaperones, and thermotolerance. J Biol Chem. 272:9086–9092. 10.1074/jbc.272.14.9086. 9083035

[jkag245-B6] Casella G et al 2019. Transcriptome signature of cellular senescence. Nucleic Acids Res. 47:7294–7305. 10.1093/nar/gkz555. PMC6698740 31251810

[jkag245-B7] Chocron ES et al 2022. Genetic and pharmacologic proteasome augmentation ameliorates Alzheimer's-like pathology in mouse and fly APP overexpression models. Sci Adv. 8:. 10.1126/sciadv.abk2252. PMC9177073 35675410

[jkag245-B8] Close GL, Kayani A, Vasilaki A, McArdle A. 2005. Skeletal muscle damage with exercise and aging. Sports Med. 35:413–427. 10.2165/00007256-200535050-00004. 15896090

[jkag245-B9] Collins GA, Goldberg AL. 2017. The logic of the 26S proteasome. Cell. 169:792–806. 10.1016/j.cell.2017.04.023. PMC5609836 28525752

[jkag245-B10] Curley M et al 2026. UBA1 knockdown dysregulates the levels of UBA1-sensitive proteins and impairs muscle function in Drosophila and mice. Dis Model Mech. 19:dmm052935. 10.1242/dmm.052935. PMC13580589 42402962

[jkag245-B11] Curley M et al 2024. Transgenic sensors reveal compartment-specific effects of aggregation-prone proteins on subcellular proteostasis during aging. Cell Rep Methods. 4:100875. 10.1016/j.crmeth.2024.100875. PMC11573793 39383859

[jkag245-B12] Dantuma NP, Lindsten K. 2010. Stressing the ubiquitin-proteasome system. Cardiovasc Res. 85:263–271. 10.1093/cvr/cvp255. 19633314

[jkag245-B13] Demontis F, Patel VK, Swindell WR, Perrimon N. 2014. Intertissue control of the nucleolus via a myokine-dependent longevity pathway. Cell Rep. 7:1481–1494. 10.1016/j.celrep.2014.05.001. PMC4125979 24882005

[jkag245-B14] Demontis F, Perrimon N. 2010. FOXO/4E-BP signaling in Drosophila muscles regulates organism-wide proteostasis during aging. Cell. 143:813–825. 10.1016/j.cell.2010.10.007. PMC3066043 21111239

[jkag245-B15] Demontis F, Piccirillo R, Goldberg AL, Perrimon N. 2013. Mechanisms of skeletal muscle aging: insights from Drosophila and mammalian models. Dis Model Mech. 6:1339–1352. 10.1242/dmm.012559. PMC3820258 24092876

[jkag245-B16] Diamant I, Clarke DJB, Evangelista JE, Lingam N, Ma'ayan A. 2025. Harmonizome 3.0: integrated knowledge about genes and proteins from diverse multi-omics resources. Nucleic Acids Res. 53:D1016–D1028. 10.1093/nar/gkae1080. PMC11701526 39565209

[jkag245-B17] Dirac-Svejstrup AB et al 2020. DDI2 is a ubiquitin-directed endoprotease responsible for cleavage of transcription factor NRF1. Mol Cell. 79:332–341.e337. 10.1016/j.molcel.2020.05.035. PMC7369636 32521225

[jkag245-B18] Durieux J, Wolff S, Dillin A. 2011. The cell-non-autonomous nature of electron transport chain-mediated longevity. Cell. 144:79–91. 10.1016/j.cell.2010.12.016. PMC3062502 21215371

[jkag245-B19] Finley D, Ozkaynak E, Varshavsky A. 1987. The yeast polyubiquitin gene is essential for resistance to high temperatures, starvation, and other stresses. Cell. 48:1035–1046. 10.1016/0092-8674(87)90711-2. 3030556

[jkag245-B20] Geier E et al 1999. A giant protease with potential to substitute for some functions of the proteasome. Science. 283:978–981. 10.1126/science.283.5404.978. 9974389

[jkag245-B21] Girondel C, Meloche S. 2021. Interleukin-17 receptor D in physiology, inflammation and cancer. Front Oncol. 11:656004. 10.3389/fonc.2021.656004. PMC8021910 33833999

[jkag245-B22] Glas R, Bogyo M, McMaster JS, Gaczynska M, Ploegh HL. 1998. A proteolytic system that compensates for loss of proteasome function. Nature. 392:618–622. 10.1038/33443. 9560160

[jkag245-B23] Goldberg AL . 2012. Development of proteasome inhibitors as research tools and cancer drugs. J Cell Biol. 199:583–588. 10.1083/jcb.201210077. PMC3494858 23148232

[jkag245-B24] Goldberg AL, Rock K. 2002. Not just research tools–proteasome inhibitors offer therapeutic promise. Nat Med. 8:338–340. 10.1038/nm0402-338. 11927937

[jkag245-B25] Graca FA et al 2022. The myokine Fibcd1 is an endogenous determinant of myofiber size and mitigates cancer-induced myofiber atrophy. Nat Commun. 13:2370. 10.1038/s41467-022-30120-1. PMC9061726 35501350

[jkag245-B26] Graca FA et al 2021. A large-scale transgenic RNAi screen identifies transcription factors that modulate myofiber size in Drosophila. PLoS Genet. 17:e1009926. 10.1371/journal.pgen.1009926. PMC8629395 34780463

[jkag245-B27] Graca FA et al 2023. Progressive development of melanoma-induced cachexia differentially impacts organ systems in mice. Cell Rep. 42:111934. 10.1016/j.celrep.2022.111934. PMC9983329 36640353

[jkag245-B28] Han ES et al 2008. The in vivo gene expression signature of oxidative stress. Physiol Genomics. 34:112–126. 10.1152/physiolgenomics.00239.2007. PMC2532791 18445702

[jkag245-B29] Hanna J, Meides A, Zhang DP, Finley D. 2007. A ubiquitin stress response induces altered proteasome composition. Cell. 129:747–759. 10.1016/j.cell.2007.03.042. 17512408

[jkag245-B30] Hanssum A et al 2014. An inducible chaperone adapts proteasome assembly to stress. Mol Cell. 55:566–577. 10.1016/j.molcel.2014.06.017. PMC4148588 25042801

[jkag245-B31] Hu JL, Todhunter ME, LaBarge MA, Gartner ZJ. 2018. Opportunities for organoids as new models of aging. J Cell Biol. 217:39–50. 10.1083/jcb.201709054. PMC5748992 29263081

[jkag245-B32] Hunt LC et al 2025a. The ubiquitin-conjugating enzyme UBE2D maintains a youthful proteome and ensures protein quality control during aging by sustaining proteasome activity. PLoS Biol. 23:e3002998. 10.1371/journal.pbio.3002998. PMC11778781 39879147

[jkag245-B33] Hunt LC et al 2019a. Circadian gene variants and the skeletal muscle circadian clock contribute to the evolutionary divergence in longevity across Drosophila populations. Genome Res. 29:1262–1276. 10.1101/gr.246884.118. PMC6673717 31249065

[jkag245-B34] Hunt LC et al 2023. An adaptive stress response that confers cellular resilience to decreased ubiquitination. Nat Commun. 14:7348. 10.1038/s41467-023-43262-7. PMC10646096 37963875

[jkag245-B35] Hunt LC et al 2021. Antagonistic control of myofiber size and muscle protein quality control by the ubiquitin ligase UBR4 during aging. Nat Commun. 12:1418. 10.1038/s41467-021-21738-8. PMC7930053 33658508

[jkag245-B36] Hunt LC et al 2025b. Proteome solubility is differentially reshaped by thermal stress and regulators of ubiquitination. J Biol Chem. 301:110517. 10.1016/j.jbc.2025.110517. PMC12391699 40713964

[jkag245-B37] Hunt LC et al 2019b. A key role for the ubiquitin ligase UBR4 in myofiber hypertrophy in Drosophila and mice. Cell Rep. 28:1268–1281.e6. 10.1016/j.celrep.2019.06.094. PMC6697171 31365869

[jkag245-B38] Hunt LC et al 2015. The glucose-sensing transcription factor MLX promotes myogenesis via myokine signaling. Genes Dev. 29:2475–2489. 10.1101/gad.267419.115. PMC4691951 26584623

[jkag245-B39] Jiao J et al 2023. Modulation of protease expression by the transcription factor Ptx1/PITX regulates protein quality control during aging. Cell Rep. 42:111970. 10.1016/j.celrep.2022.111970. PMC9933915 36640359

[jkag245-B40] Jiao J, Demontis F. 2017. Skeletal muscle autophagy and its role in sarcopenia and organismal aging. Curr Opin Pharmacol. 34:1–6. 10.1016/j.coph.2017.03.009. 28407519

[jkag245-B41] Johnsen O, Murphy P, Prydz H, Kolsto AB. 1998. Interaction of the CNC-bZIP factor TCF11/LCR-F1/Nrf1 with MafG: binding-site selection and regulation of transcription. Nucleic Acids Res. 26:512–520. 10.1093/nar/26.2.512. PMC147270 9421508

[jkag245-B42] Jovaisaite V, Mouchiroud L, Auwerx J. 2014. The mitochondrial unfolded protein response, a conserved stress response pathway with implications in health and disease. J Exp Biol. 217:137–143. 10.1242/jeb.090738. PMC3867496 24353213

[jkag245-B43] Kageyama S et al 2014. Proteasome dysfunction activates autophagy and the Keap1-Nrf2 pathway. J Biol Chem. 289:24944–24955. 10.1074/jbc.M114.580357. PMC4155663 25049227

[jkag245-B44] Kamens JL et al 2023. Proteasome inhibition targets the KMT2A transcriptional complex in acute lymphoblastic leukemia. Nat Commun. 14:809. 10.1038/s41467-023-36370-x. PMC9925443 36781850

[jkag245-B45] Keck S, Nitsch R, Grune T, Ullrich O. 2003. Proteasome inhibition by paired helical filament-tau in brains of patients with Alzheimer's disease. J Neurochem. 85:115–122. 10.1046/j.1471-4159.2003.01642.x. 12641733

[jkag245-B46] Kelley LP et al 2025. Integrated analysis of transcriptional and metabolic responses to mitochondrial stress. Cell Rep Methods. 5:101027. 10.1016/j.crmeth.2025.101027. PMC12256945 40233762

[jkag245-B47] Kimura Y et al 2009. An inhibitor of a deubiquitinating enzyme regulates ubiquitin homeostasis. Cell. 137:549–559. 10.1016/j.cell.2009.02.028. 19410548

[jkag245-B48] Kisselev AF, Goldberg AL. 2001. Proteasome inhibitors: from research tools to drug candidates. Chem Biol. 8:739–758. 10.1016/S1074-5521(01)00056-4. 11514224

[jkag245-B49] Koizumi S et al 2016. The aspartyl protease DDI2 activates Nrf1 to compensate for proteasome dysfunction. Elife. 5:e18357. 10.7554/eLife.18357. PMC5001836 27528193

[jkag245-B50] Kourtis N, Tavernarakis N. 2011. Cellular stress response pathways and ageing: intricate molecular relationships. Embo J. 30:2520–2531. 10.1038/emboj.2011.162. PMC3155297 21587205

[jkag245-B51] Lee CS et al 2004. A proteasomal stress response: pre-treatment with proteasome inhibitors increases proteasome activity and reduces neuronal vulnerability to oxidative injury. J Neurochem. 91:996–1006. 10.1111/j.1471-4159.2004.02813.x. 15525353

[jkag245-B52] Lee DH, Goldberg AL. 1998. Proteasome inhibitors cause induction of heat shock proteins and trehalose, which together confer thermotolerance in *Saccharomyces cerevisiae*. Mol Cell Biol. 18:30–38. 10.1128/MCB.18.1.30. PMC121446 9418850

[jkag245-B53] Lehallier B et al 2019. Undulating changes in human plasma proteome profiles across the lifespan. Nat Med. 25:1843–1850. 10.1038/s41591-019-0673-2. PMC7062043 31806903

[jkag245-B54] Lehrbach NJ, Breen PC, Ruvkun G. 2019. Protein sequence editing of SKN-1A/Nrf1 by peptide:N-glycanase controls proteasome gene expression. Cell. 177:737–750.e715. 10.1016/j.cell.2019.03.035. PMC6574124 31002798

[jkag245-B55] Lehrbach NJ, Ruvkun G. 2016. Proteasome dysfunction triggers activation of SKN-1A/Nrf1 by the aspartic protease DDI-1. Elife. 5:e17721. 10.7554/eLife.17721. PMC4987142 27528192

[jkag245-B56] Lipinszki Z et al 2013. A novel interplay between the ubiquitin-proteasome system and serine proteases during Drosophila development. Biochem J. 454:571–583. 10.1042/BJ20130040. 23805892

[jkag245-B57] Lundgren J, Masson P, Mirzaei Z, Young P. 2005. Identification and characterization of a Drosophila proteasome regulatory network. Mol Cell Biol. 25:4662–4675. 10.1128/MCB.25.11.4662-4675.2005. PMC1140619 15899868

[jkag245-B58] Lv Y et al 2020. The role of PSMB5 in sodium arsenite-induced oxidative stress in L-02 cells. Cell Stress Chaperones. 25:533–540. 10.1007/s12192-020-01104-1. PMC7192974 32301004

[jkag245-B59] Marciniak SJ, Ron D. 2006. Endoplasmic reticulum stress signaling in disease. Physiol Rev. 86:1133–1149. 10.1152/physrev.00015.2006. 17015486

[jkag245-B60] Morton JP, Kayani AC, McArdle A, Drust B. 2009. The exercise-induced stress response of skeletal muscle, with specific emphasis on humans. Sports Med. 39:643–662. 10.2165/00007256-200939080-00003. 19769414

[jkag245-B61] Munch C . 2018. The different axes of the mammalian mitochondrial unfolded protein response. BMC Biol. 16:81. 10.1186/s12915-018-0548-x. PMC6060479 30049264

[jkag245-B62] Murray JI et al 2004. Diverse and specific gene expression responses to stresses in cultured human cells. Mol Biol Cell. 15:2361–2374. 10.1091/mbc.e03-11-0799. PMC404029 15004229

[jkag245-B63] Obeng EA et al 2006. Proteasome inhibitors induce a terminal unfolded protein response in multiple myeloma cells. Blood. 107:4907–4916. 10.1182/blood-2005-08-3531. PMC1895817 16507771

[jkag245-B64] Ogborn DI, McKay BR, Crane JD, Parise G, Tarnopolsky MA. 2014. The unfolded protein response is triggered following a single, unaccustomed resistance-exercise bout. Am J Physiol Regul Integr Comp Physiol. 307:R664–R669. 10.1152/ajpregu.00511.2013. 25009220

[jkag245-B65] Owusu-Ansah E, Song W, Perrimon N. 2013. Muscle mitohormesis promotes longevity via systemic repression of insulin signaling. Cell. 155:699–712. 10.1016/j.cell.2013.09.021. PMC3856681 24243023

[jkag245-B66] Patel VK, Demontis F. 2014. GDF11/myostatin and aging. Aging (Albany NY). 6:351–352. 10.18632/aging.100666. PMC4069261 24902527

[jkag245-B67] Piccirillo R, Goldberg AL. 2012. The p97/VCP ATPase is critical in muscle atrophy and the accelerated degradation of muscle proteins. EMBO J. 31:3334–3350. 10.1038/emboj.2012.178. PMC3411080 22773186

[jkag245-B68] Qian X et al 2016. Brain-region-specific organoids using mini-bioreactors for modeling ZIKV exposure. Cell. 165:1238–1254. 10.1016/j.cell.2016.04.032. PMC4900885 27118425

[jkag245-B69] Radhakrishnan SK, den Besten W, Deshaies RJ. 2014. p97-dependent retrotranslocation and proteolytic processing govern formation of active Nrf1 upon proteasome inhibition. Elife. 3:e01856. 10.7554/eLife.01856. PMC3896944 24448410

[jkag245-B70] Radhakrishnan SK et al 2010. Transcription factor Nrf1 mediates the proteasome recovery pathway after proteasome inhibition in mammalian cells. Mol Cell. 38:17–28. 10.1016/j.molcel.2010.02.029. PMC2874685 20385086

[jkag245-B71] Rai M et al 2025a. Perturbation of multiprotein complexes in skeletal muscle induces protective proteases in the CNS that degrade pathogenic proteins. NPJ Aging. 11:97. 10.1038/s41514-025-00288-z. PMC12660746 41309669

[jkag245-B72] Rai M et al 2021a. Proteasome stress in skeletal muscle mounts a long-range protective response that delays retinal and brain aging. Cell Metab. 33:1137–1154.e1139. 10.1016/j.cmet.2021.03.005. PMC8172468 33773104

[jkag245-B73] Rai M, Curley M, Coleman Z, Demontis F. 2022. Contribution of proteases to the hallmarks of aging and to age-related neurodegeneration. Aging Cell. 21:e13603. 10.1111/acel.13603. PMC9124314 35349763

[jkag245-B74] Rai M et al 2021b. Analysis of proteostasis during aging with western blot of detergent-soluble and insoluble protein fractions. STAR Protoc. 2:100628. 10.1016/j.xpro.2021.100628. PMC8246638 34235493

[jkag245-B75] Rai M, Demontis F. 2022. Muscle-to-brain signaling via myokines and myometabolites. Brain Plast. 8:43–63. 10.3233/BPL-210133. PMC9661353 36448045

[jkag245-B76] Rai M, Hunt LC, Demontis F. 2025b. Stress responses induced by perturbation of the ubiquitin-proteasome system. Trends Biochem Sci. 50:175–178. 10.1016/j.tibs.2024.12.011. PMC11890940 39779413

[jkag245-B77] Robles-Murguia M, Hunt LC, Finkelstein D, Fan Y, Demontis F. 2019. Tissue-specific alteration of gene expression and function by RU486 and the GeneSwitch system. NPJ Aging Mech Dis. 5:6. 10.1038/s41514-019-0036-8. PMC6529481 31123597

[jkag245-B78] Robles-Murguia M et al 2020. Muscle-derived Dpp regulates feeding initiation via endocrine modulation of brain dopamine biosynthesis. Genes Dev. 34:37–52. 10.1101/gad.329110.119. PMC6938663 31831628

[jkag245-B79] Rouillard AD et al 2016. The harmonizome: a collection of processed datasets gathered to serve and mine knowledge about genes and proteins. Database (Oxford). 2016:baw100. 10.1093/database/baw100. PMC4930834 27374120

[jkag245-B80] Saul D et al 2022. A new gene set identifies senescent cells and predicts senescence-associated pathways across tissues. Nat Commun. 13:4827. 10.1038/s41467-022-32552-1. PMC9381717 35974106

[jkag245-B81] Schmidt M, Finley D. 2014. Regulation of proteasome activity in health and disease. Biochim Biophys Acta. 1843:13–25. 10.1016/j.bbamcr.2013.08.012. PMC3858528 23994620

[jkag245-B82] Sha Z, Goldberg AL. 2014. Proteasome-mediated processing of Nrf1 is essential for coordinate induction of all proteasome subunits and p97. Curr Biol. 24:1573–1583. 10.1016/j.cub.2014.06.004. PMC4108618 24998528

[jkag245-B83] Sha Z, Schnell HM, Ruoff K, Goldberg A. 2018. Rapid induction of p62 and GABARAPL1 upon proteasome inhibition promotes survival before autophagy activation. J Cell Biol. 217:1757–1776. 10.1083/jcb.201708168. PMC5940303 29535191

[jkag245-B84] Steffen J, Seeger M, Koch A, Kruger E. 2010. Proteasomal degradation is transcriptionally controlled by TCF11 via an ERAD-dependent feedback loop. Mol Cell. 40:147–158. 10.1016/j.molcel.2010.09.012. 20932482

[jkag245-B85] Stephan A, Graca FA, Hunt LC, Demontis F. 2022. Electroporation of small interfering RNAs into tibialis anterior muscles of mice. Bio Protoc. 12:e4428. 10.21769/BioProtoc.4428. PMC9244496 35799907

[jkag245-B86] Stephan A et al 2026. Insolubilome profiling defines molecular features that influence protein insolubility with aging. Dev Cell. 61:936–958.e7. 10.1016/j.devcel.2026.01.015. 41720097

[jkag245-B87] Tabula Muris C . 2020. A single-cell transcriptomic atlas characterizes ageing tissues in the mouse. Nature. 583:590–595. 10.1038/s41586-020-2496-1. PMC8240505 32669714

[jkag245-B88] Tang D et al 2023. SRplot: a free online platform for data visualization and graphing. PLoS One. 18:e0294236. 10.1371/journal.pone.0294236. PMC10635526 37943830

[jkag245-B89] Taylor RC, Berendzen KM, Dillin A. 2014. Systemic stress signalling: understanding the cell non-autonomous control of proteostasis. Nat Rev Mol Cell Biol. 15:211–217. 10.1038/nrm3752. PMC5922984 24556842

[jkag245-B90] Taylor RC, Dillin A. 2013. XBP-1 is a cell-nonautonomous regulator of stress resistance and longevity. Cell. 153:1435–1447. 10.1016/j.cell.2013.05.042. PMC4771415 23791175

[jkag245-B91] Tsakiri EN et al 2019. Proteasome dysfunction induces excessive proteome instability and loss of mitostasis that can be mitigated by enhancing mitochondrial fusion or autophagy. Autophagy 15:1757–1773. 10.1080/15548627.2019.1596477. PMC6735541 31002009

[jkag245-B92] Tseng BP, Green KN, Chan JL, Blurton-Jones M, LaFerla FM. 2008. Abeta inhibits the proteasome and enhances amyloid and tau accumulation. Neurobiol Aging. 29:1607–1618. 10.1016/j.neurobiolaging.2007.04.014. PMC2664168 17544172

[jkag245-B93] Tsvetkov P et al 2015. Compromising the 19S proteasome complex protects cells from reduced flux through the proteasome. Elife. 4: e08467. 10.7554/eLife.08467. PMC4551903 26327695

[jkag245-B94] van den Boom J, Meyer H. 2018. VCP/p97-mediated unfolding as a principle in protein homeostasis and signaling. Mol Cell. 69:182–194. 10.1016/j.molcel.2017.10.028. 29153394

[jkag245-B95] van Oosten-Hawle P, Porter RS, Morimoto RI. 2013. Regulation of organismal proteostasis by transcellular chaperone signaling. Cell. 153:1366–1378. 10.1016/j.cell.2013.05.015. PMC3955170 23746847

[jkag245-B96] Wang CY et al 2017. PSMB5 plays a dual role in cancer development and immunosuppression. Am J Cancer Res. 7:2103–2120. PMC5714741 29218236

[jkag245-B97] Wang D et al 2021. GDF15: emerging biology and therapeutic applications for obesity and cardiometabolic disease. Nat Rev Endocrinol. 17:592–607. 10.1038/s41574-021-00529-7. 34381196

[jkag245-B98] Wang EW et al 2000. Integration of the ubiquitin-proteasome pathway with a cytosolic oligopeptidase activity. Proc Natl Acad Sci U S A. 97:9990–9995. 10.1073/pnas.180328897. PMC27648 10954757

[jkag245-B99] Wang H et al 2025. CRESTA: a comprehensive transcriptome atlas for cellular response to external stressors. Nucleic Acids Res. 54:D1005–D1016. 10.1093/nar/gkaf968. PMC12807750 41024715

[jkag245-B100] Wojcik C, DeMartino GN. 2002. Analysis of Drosophila 26 S proteasome using RNA interference. J Biol Chem. 277:6188–6197. 10.1074/jbc.M109996200. 11739392

[jkag245-B101] Xie Y, Varshavsky A. 2001. RPN4 is a ligand, substrate, and transcriptional regulator of the 26S proteasome: a negative feedback circuit. Proc Natl Acad Sci U S A. 98:3056–3061. 10.1073/pnas.071022298. PMC30606 11248031

