# Supplementary material for: Analysis of proteasome stress in different cell types defines a conserved transcriptional response signature and its modulation with aging
Source: G3 (Bethesda). 2026 Sep 16;16(11):jkag245. doi: 10.1093/g3journal/jkag245 (PMC13732486; doi:10.1093/g3journal/jkag245)
Supplement: Supplementary file 1 [file jkag245_supplementary_data.zip › Supplementary_Figures_and_Dataset_Legends_G3-2026-407182.pdf]

# SUPPLEMENTARY INFORMATION

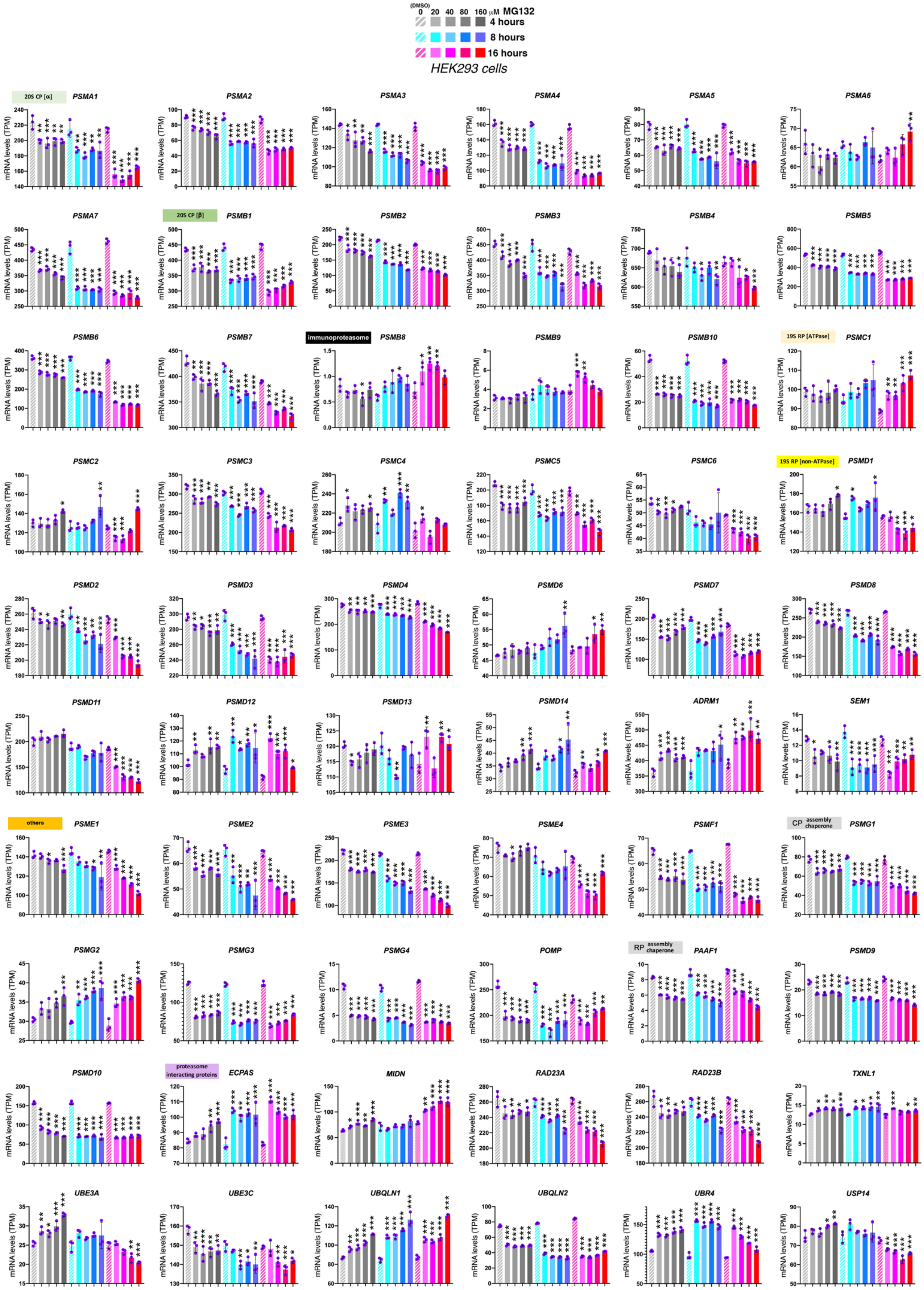
(DMSO)
0 20 40 80 160 μM MG132
4 hours
8 hours
16 hours
HEK293 cells
mRNA levels (TPM)
20S CP [α]
PSMA1
PSMA2
PSMA3
PSMA4
PSMA5
PSMA6
PSMA7
20S CP [β]
PSMB1
PSMB2
PSMB3
PSMB4
PSMB5
PSMB6
PSMB7
immunoproteasome
PSMB8
PSMB9
PSMB10
19S RP [ATPase]
PSMC1
PSMC2
PSMC3
PSMC4
PSMC5
PSMC6
19S RP [non-ATPase]
PSMD1
PSMD2
PSMD3
PSMD4
PSMD6
PSMD7
PSMD8
PSMD11
PSMD12
PSMD13
PSMD14
ADRM1
SEM1
others
PSME1
PSME2
PSME3
PSME4
PSMF1
CP assembly chaperone
PSMG1
PSMG2
PSMG3
PSMG4
POMP
RP assembly chaperone
PAAF1
PSMD9
PSMD10
proteasome interacting proteins
ECPAS
MIDN
RAD23A
RAD23B
TXNL1
UBE3A
UBE3C
UBQLN1
UBQLN2
UBR4
USP14

**Supplementary Figure 1.** Transcriptional modulation of proteasome components in response to time-dose treatment of HEK293T cells with MG132. Human HEK293T cells were treated with different concentrations of the proteasome inhibitor MG132 (20, 40, 80, 160 μM) compared to the vehicle control (DMSO) for 4, 8, or 16 hours (n=3 biological replicates/group). The graphs display the mRNA levels (TPM values) of proteasome components of the 20S catalytic core (α and β subunits), immunoproteasome subunits, ATPase and non-ATPase components of the 19S regulatory particle, other components (e.g., PSME subunits), assembly chaperones of the core particle (CP) and regulatory particle (RP), and proteasome interacting proteins (the first protein in each of these categories is indicated). The graphs report the mean ±SD with n=3 biological replicates/group. The statical analyses refer to the comparison of each sample to the corresponding DMSO control for the same length of treatment (one-way ANOVA with *$P$<0.05, **$P$<0.01, and ***$P$<0.001). Overall, MG132 regulates in a divergent manner the expression of different proteasome components. Specifically, in each category, some proteasome components are upregulated whereas others are downregulated.

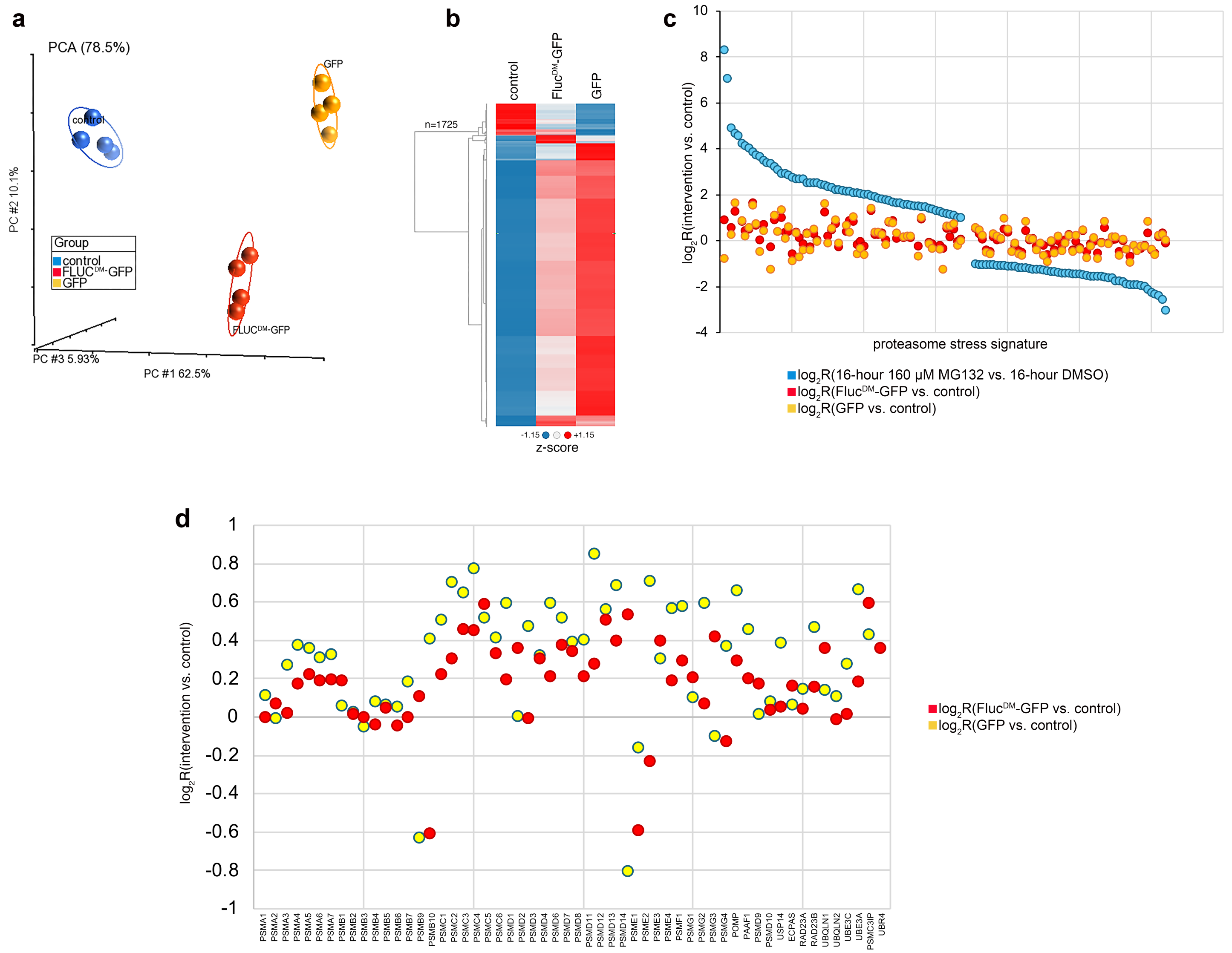


**Supplementary Figure 2. Transcriptional changes induced by GFP and the misfolding-prone protein Fluc$^{DM}$-GFP.** Transfection of human HEK293T cells with plasmids for the expression of GFP and Fluc$^{DM}$-GFP, a misfolding-prone firefly luciferase.

a) PCA analyses indicate that GFP and Fluc$^{DM}$-GFP induce many transcriptional changes compared to control cells (n=4 biological replicates/group).

b) Hierarchical clustering of the 1725 genes that are most highly regulated ($log_2R>|1|$ and $P<0.05$ in at least one comparison) by GFP and Fluc$^{DM}$-GFP vs. control cells. The z-scores were used to visualize upregulated (red) and downregulated (blue) genes but not to determine differential expressions.

c) Modulation of the proteasome stress signature by GFP and Fluc$^{DM}$-GFP. The *y*-axis displays the changes ($log_2R$) induced by an intervention vs. control, whereas the *x*-axis displays the genes that are significantly and consistently upregulated and downregulated by proteasome inhibition via MG132 across multiple cell types (proteasome stress signature; Figure 5). Modulation of the proteasome stress signature in HEK293T cells minimally coincides with the gene expression changes induced by GFP and Fluc$^{DM}$-GFP. These findings suggest that perturbation of proteostasis by an abundant protein (GFP) or a misfolding-prone protein (Fluc$^{DM}$-GFP) does not necessarily induce a proteasome stress response. See also Supplementary Dataset 6.

d) Modulation of proteasome gene expression by GFP and Fluc$^{DM}$-GFP. The *y*-axis displays the changes ($log_2R$) induced by an intervention vs. control, whereas the *x*-axis displays the different components of the proteasome (ordered as in Fig. 4). There is a limited modulation of proteasome gene expression that occurs similarly in response to either GFP or Fluc$^{DM}$-GFP.

## SUPPLEMENTARY DATASETS

**Supplementary Dataset 1.** RNA-seq of human HEK293T cells treated with different time-doses of MG132 or with siRNAs for components of the catalytic core of the proteasome, compared to controls.

**Supplementary Dataset 2.** RNA-seq of mouse C2C12 myotubes treated with different doses of MG132 vs. DMSO controls.

**Supplementary Dataset 3.** RNA-seq of mouse C2C12 myoblasts treated with siRNAs for components of the catalytic core of the proteasome vs. controls.

**Supplementary Dataset 4.** RNA-seq of human brain (cortical) organoids treated with MG132 vs. DMSO controls.

**Supplementary Dataset 5.** The transcriptional signature of proteasome stress.

**Supplementary Dataset 6.** RNA-seq of human HEK293T cells transfected with plasmids encoding for $Fluc^{DM}$-GFP, GFP, and controls.

**Supplementary Dataset 7.** Source Data file.
